# Natural Products Produced in Culture by Biosynthetically Talented *Salinispora arenicola* Strains Isolated from Northeastern and South Pacific Marine Sediments

**DOI:** 10.3390/molecules27113569

**Published:** 2022-06-02

**Authors:** David E. Williams, Kalindi D. Morgan, Doralyn S. Dalisay, Teatulohi Matainaho, Elodie Perrachon, Noemie Viller, Maïlys Delcroix, Jeanne Gauchot, Haruka Niikura, Brian O. Patrick, Katherine S. Ryan, Raymond J. Andersen

**Affiliations:** 1 Departments of Chemistry and Earth, Ocean and Atmospheric Sciences, University of British Columbia, Vancouver, BC V6T 1Z1, Canada; davewill@chem.ubc.ca (D.E.W.); kalindi.morgan@unbc.ca (K.D.M.); elodie.perrachon@ensiacet.fr (E.P.); noemie.viller@ensiacet.fr (N.V.); mailys.delcroix@ensiacet.fr (M.D.); jeanne.gauchot@ensiacet.fr (J.G.); 2 Department of Chemistry, University of British Columbia, Vancouver, BC V6T 1Z1, Canada; harukaniikura13826@gmail.com (H.N.); bpatrick@chem.ubc.ca (B.O.P.); 3 Department of Chemistry and Biochemistry, University of Northern British Columbia, Prince George, BC V2N 4Z9, Canada; 4 Center for Chemical Biology and Biotechnology (C2B2), Department of Biology, College of Liberal Arts, Sciences and Education, University of San Agustin, Iloilo 5000, Philippines; ddalisay@usa.edu.ph; 5 School of Medicine and Health Sciences, University of Papua New Guinea, Boroko P.O. Box 5623, NCD, Papua New Guinea; lmatainaho@yahoo.com

**Keywords:** bacteria, marine, sediments, *Salinispora*, polyketide, Pacific Ocean

## Abstract

Laboratory cultures of two ‘biosynthetically talented’ bacterial strains harvested from tropical and temperate Pacific Ocean sediment habitats were examined for the production of new natural products. Cultures of the tropical *Salinispora arenicola* strain RJA3005, harvested from a PNG marine sediment, produced salinorcinol (**3**) and salinacetamide (**4**), which had previously been reported as products of engineered and mutated strains of *Amycolatopsis mediterranei*, but had not been found before as natural products. An *S*. *arenicola* strain RJA4486, harvested from marine sediment collected in the temperate ocean waters off British Columbia, produced the new aminoquinone polyketide salinisporamine (**5**). Natural products **3**, **4**, and **5** are putative shunt products of the widely distributed rifamycin biosynthetic pathway.

## 1. Introduction

**Figure 1:**
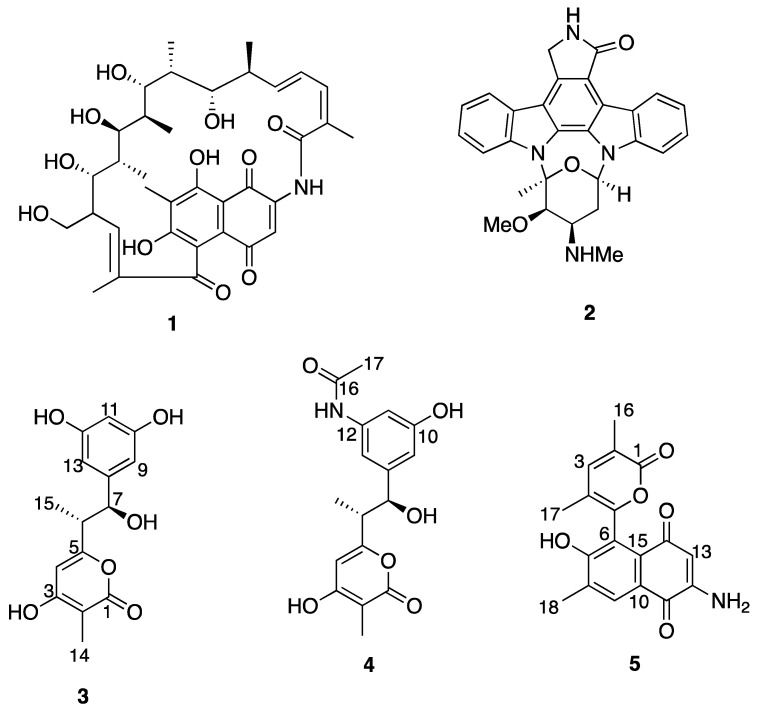
Chemical structures of compounds **1** to **5**.

Marine isolates of bacteria in the genera *Salinispora* have proven to be a rich source of novel natural products that often exhibit biological activities of interest for drug development [1,2]. They have also been found to be an excellent resource for exploring the ‘one strain many compounds’ (OSMAC) strategy for bioactive natural product discovery [3], whereby culture conditions are varied to elicit the production of new compounds. As part of our ongoing interest in biologically active natural products produced by *Salinispora arenicola* obtained from both tropical and temperate Pacific Ocean marine habitats, we have discovered two biosynthetically talented isolates RJA3005 and RJA4486. Isolate RJA3005 is a strain of *Salinispora* *arenicola*, obtained from marine sediment collected in Papua New Guinea, while isolate RJA4486 is a strain of *S. arenicola* harvested from marine sediment collected from the temperate ocean waters off the coast of British Columbia. The tropical *S. arenicola* strain RJA3005 attracted our attention because its crude extract was active in a bioassay screen for phosphatase inhibitors, while the temperate water *S. arenicola* strain RJA4486 represented a range extension for *S. arenicola* [4,5] and it produced the two known bioactive natural products rifamycin W (**1**) [6] and staurosporine (**2**) [7] (Figure 1).

Prompted by their demonstrated capabilities to produce bioactive natural products and bioactive crude extracts, we have further interrogated isolates RJA3005, and RJA4486 by varying the culture conditions or investigating very minor metabolites in an attempt to uncover new natural products from their cultures. Bioassay-guided efforts to isolate a compound responsible for the phosphatase inhibitory activity exhibited by extracts of an *S. arenicola* strain RJA3005 culture failed to identify an active natural product. However, as part of this exercise, we discovered the very minor metabolites salinorcinol (**3**) and salinacetamide (**4**). Compound **3** has been reported as the product of feeding an engineered *Amycolatopsis mediterranei* S699 strain (-AHBA synthase) with synthetic aromatic precursors [8], and compound **4** has been reported as a shunt product of an engineered *A. mediterranei* S699 strain [9], but neither **3** nor **4** has been reported as a natural product produced by a wild-type bacteria in culture. Investigation of very minor metabolites with unusual UV spectra in the organic extract of cultures of *S. arenicola* strain RJA4486 resulted in the isolation of the aminoquinone salinisporamine (**5**). Herein, we describe the isolation and structure elucidation of the new microbial natural products **3**, **4**, and **5** (Figure 1).

## 2. Results

*S. arenicola* strain RJA3005 (16S rRNA gene sequence, GenBank accession no. OM728180) was grown as lawns on MM1 solid agar prepared with seawater, and the mature bacterial lawns and agar were cut into small squares and jointly extracted by soaking in multiple batches of EtOAc. The combined EtOAc extracts were concentrated in vacuo and then purified via sequential application of Sephadex LH20 chromatography, step-gradient C_18_ reversed phase flash chromatography, and C_18_ reversed-phase HPLC to give pure samples of salinorcinol (**3**) and salinacetamide (**4**).

**Figure 2:**
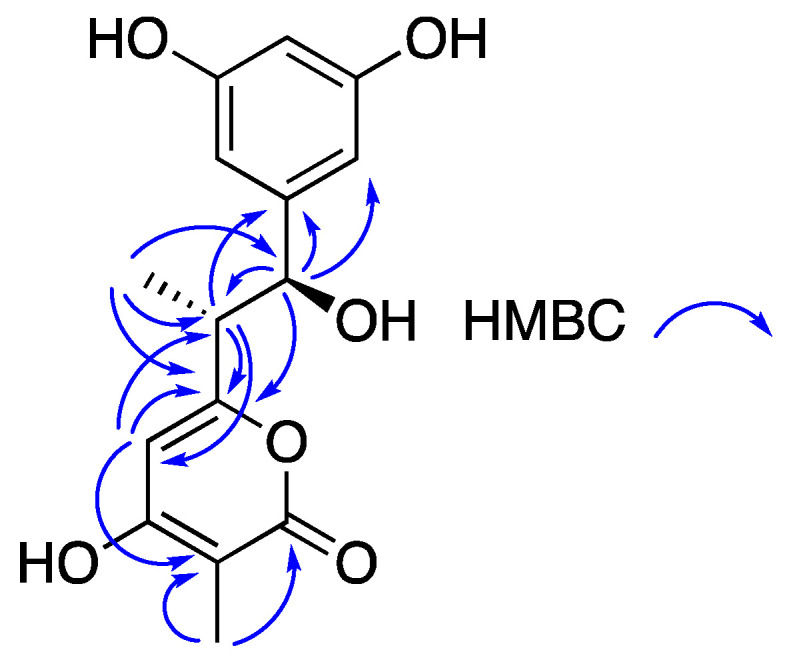
Selected HMBC correlations observed for salinorcinol (**3**).

Compound **3** gave a [M + H]^+^ ion in the HRESIMS at *m/z* 293.1046 appropriate for a molecular formula of C15H16O6 that requires eight sites of unsaturation. The proton NMR spectrum of **3** (Table 1) contained a resonance at δ_H_ 6.16 (H-9/H-13) meta coupled (*J* = 2.2 Hz) to a resonance at δ_H_ 6.09 (H-11), that identified a symmetrical 1,3,5 trisubstituted benzene ring. HMBC correlations (Figure 2) were observed between the proton resonances at δ_H_ 6.16 (H-9/H-13)), 6.09 (H-11), and 9.15 (OH-10/OH-12), and a carbon resonance at δ_c_ 157.9 (C-10/C-12), and between the proton resonance at δ_H_ 6.16 (H-9/H-13) and carbon resonances at δ_c_ 104.8 (C-9/C-13), 101.5 (C-11), and 74.7 (C-7) consistent with a 1 alkyl, 3,5 dihydroxy benzene substructure. A methine singlet at δ_H_ 5.99 (H-4; δ_c_ 100.0) showed HMBC correlations into non-protonated carbon resonances at δ_c_ 164.2 (C-5), 164.9 (C-3), and δ_c_ 96.6 (C-2), and a methyl singlet at δ_H_ 1.75 (δ_c_ 8.5, Me-14) showed HMBC correlations into carbon resonances at δ_c_ 165.2 (C-3) and 96.6 (C-2). This set of resonances was assigned to a 2-methyl, 3-hydroxy, 5-alkyl pyrone substructure (C-1 to C-5/Me-14). COSY correlations identified a three-carbon spin system consisting of a methyl (δ_H_ 0.85, Me-15), an allylic methine (δ_H_ 2.66, H-6), and an oxymethine (δ_H_ 4.58, H-7) attached to an alcohol (δ_H_ 5.32, OH-7). HMBC correlations between the methyl resonance at δ_H_ 0.85 (Me-15) and the allylic methine carbon resonance at δ_c_ 45.4 (C-6) and the oxymethine carbon resonance at δ_c_ 74.7 (C-7) confirmed the identity of the three-carbon aliphatic fragment.

The three identified substructures accounted for all of the atoms and sites of unsaturation required by the molecular formula of **3**. HMBC correlations (Figure 2) between the methyl doublet at δ_H_ 0.85 (Me-15) and the carbon resonance at δ_c_ 164.2 (C-5) and between the allylic methine at 2.66 (H-6) and the carbon resonances at δ_c_ 164.2 (C-5) and 100.0 (C-4) connected the allylic carbon (C-6) to the pyrone, and HMBC correlations between the oxy-methine resonance at δ_H_ 4.58 (H-7) and the dihydroxybenzene ring carbon resonances at δ_C_ 145.5 (C-8) and 104.8 (C-9/C-13) linked the oxymethine carbon to the benzene ring at C-8, completing the constitution of **3**.

The natural product **3** has the same constitution as a compound produced by a mutated rifamycin producer *A. mediterranei* S699 (-AHBA synthase) that had been fed 3,5-dihydroxybenzoic acid [8]. Our discovery of **3** in extracts of *S. arenicola* strain RJA3005 cultures is the first report of **3** as a natural product from a wild-type bacterial culture, and indeed as an entirely biosynthesized molecule. We have named the natural product salinorcinol (**3**). The absolute configurations at C-6 and C-7 in the semisynthetic bioengineered sample of **3** were assigned as 6*R*,7*S*. The chemical shifts at C-6/H-6 and C-7/H-7, as well as the ^1^H/^1^H coupling constants between H-6 and H-7 in our natural product and the engineered compound are virtually identical. Therefore, we assume the natural product **3** is also 6*R*,7*S*-salinorcinol (**3**).

Salinacetamide (**4**) gave a [M + H]^+^ ion in the HRESIMS at *m/z* 334.1297 appropriate for a molecular formula of C_17_H_19_O_6_N that requires 9 sites of unsaturation, differing from that of salinorcinol (**3**) by the addition of C_2_H_3_N. Comparison of the NMR data of **4** and **3** (see Table 1 and Supplementary Materials) revealed a loss of symmetry in the 1,3,5 tri-substitution about the benzene ring of **4**. In all other respects, **4** and **3** were structurally identical. An NH resonance at δ_H_ 9.80 in the HMBC spectrum of **4** showed correlations to carbonyl resonating at δ_C_ 168.3 (C-16) and an aromatic carbon at δ_C_ 140.3 (C), that was assigned to C-12. A methyl singlet at δ_H_ 2.04 (C-17, δ_C_ 24.2) was also correlated in the HMBC spectrum to the carbonyl at δ_C_ 168.3. The placement of an acetamide functionality at C-12 was consistent with both the NMR and MS data obtained for salinacetamide (**4**). A compound isolated from cultures of a rifamycin producer *A. mediterranei* S699 that was subjected to mutations was assigned the constitution of **4** solely based on HPLC MS data. Two of the *A. mediterranei* S699 mutations involved the loss of a 21kb DNA fragment [8,9] of the rifamycin gene cluster’s post-PKS modification genes [10] and a mutation involving a *rifF* deletion [9]. The samples of **4** identified from cultures of the mutant strains were never fully characterized by NMR analysis and, to the best of our knowledge, **4** has not been reported as a natural product from cultures of a wild-type bacterium.

**Figure 3:**
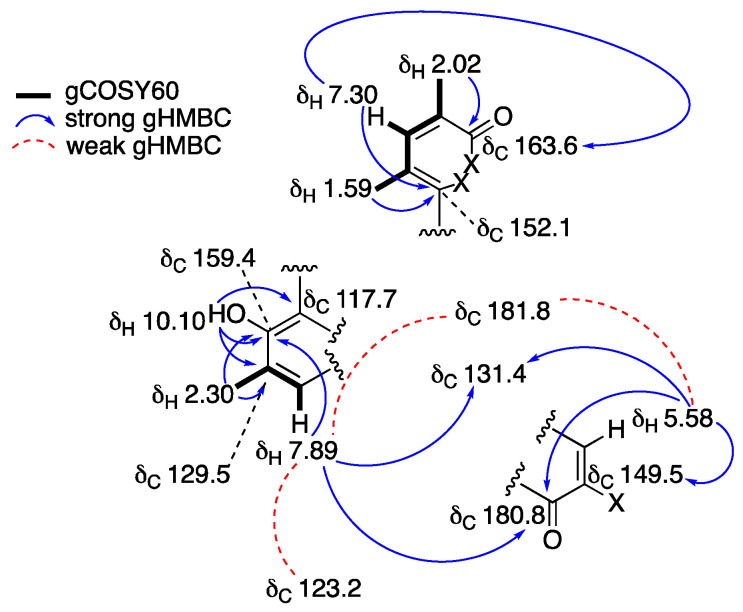
Selected COSY60 and HMBC correlations observed for salinisporamine (**5**).

**Figure 4:**
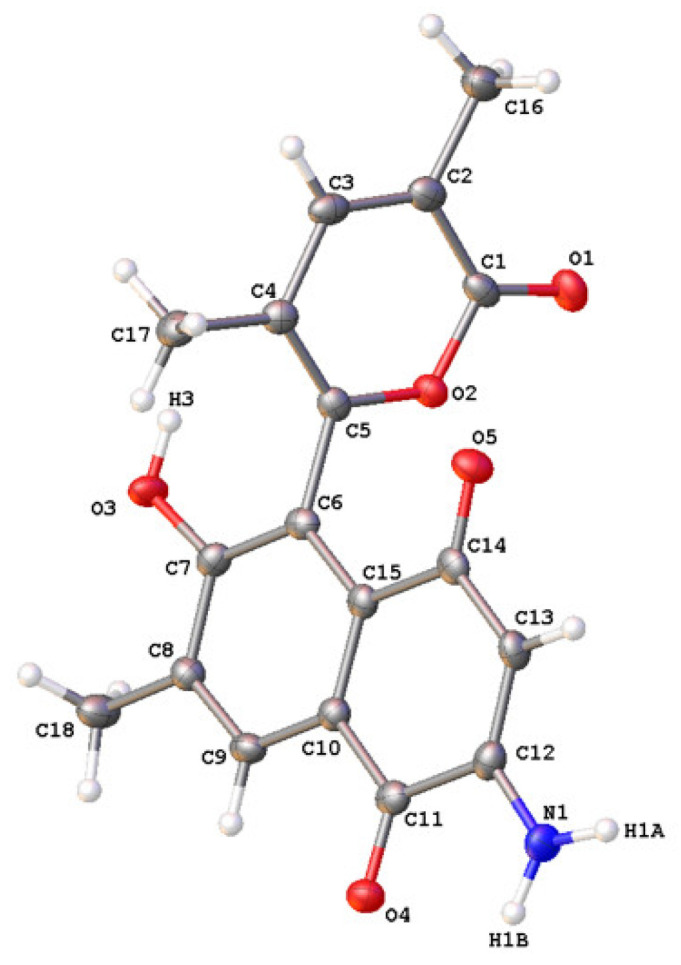
ORTEP-style diagram for salinisporamine (**5**) drawn with 50% probability ellipsoids.

Cultures of the Northeastern Pacific *S. arenicola* strain RJA4486 (16S rRNA gene sequence, GenBank accession no. OM721757) were grown as lawns on solid agar containing marine medium and the mature cultures were extracted with EtOAc as described above. The EtOAc extracts of the combined cells and solid agar media were fractionated using sequential application of Sephadex LH20 chromatography, step-gradient Si Gel flash chromatography, and C_18_ reversed-phase HPLC to give a pure salinisporamine (**5**) as optically inactive blade-shaped orange crystals with a complex UV spectrum (λ_max_ at 195, 217, 282 and 322 nm). Salinisporamine (**5**) gave a [M + H]^+^ ion in the HRESIMS at *m/z* 326.1022 appropriate for a molecular formula of C_18_H_15_NO_5_ that requires 12 sites of unsaturation. In the presence of residual TFA, **5** gave a sharp well-resolved peak when analyzed by C_18_ reversed-phase HPLC using a variety of solvent systems and it gave a single clean molecular ion in the HRESIMS. Despite the HPLC and MS indications of purity, all of the resonances in the ^1^H NMR spectrum of **5** recorded in DMSO-*d_6_* were doubled (Supporting Information). When repurified by HPLC with no TFA present, the resulting ^1^H NMR spectrum of **5** recorded in DMSO-*d_6_* showed only a single set of well-resolved resonances (Table 2, Supplementary Materials). Detailed analysis of the 1D and 2D NMR data (Table 2, Figure 3) revealed that the structure of **5** consisted of a highly functionalized aromatic ring system with extended conjugation into perhaps a quinone moiety as characteristic carbonyl resonances were observed at δ_C_ 180.8 (C-11) and 181.8 (C-14). In addition, ^1^H NMR resonances at δ_H_ 2.30 (Me-18), 2.02 (Me-16), and 1.59 (Me-17), that each integrated for three protons, and correlated to carbons at δ_C_ 16.7 (C-18), 16.2 (C-16) and 14.7 (C-17), respectively, in the HSQC experiment suggested that the structure of **5** possessed 3 aromatic and/or olefinic methyl residues. Three aromatic or olefinic methine carbons (δ_C/H_: 144.2/7.30 (C-3); 130.5/7.89 (C-9), 102.6/5.58 (C-13)) were also observed in the NMR data along with a phenolic proton resonance at δ_H_ 10.10 (OH-7). Figure 3 illustrates the three structural fragments of **5** that could be assigned from the NMR data. However, the complete constitution of **5** could not be elucidated from the NMR data alone. Therefore, crystals of **5** were subjected to single-crystal X-ray diffraction analysis and the resulting ORTEP-style diagram in Figure 4 shows the complete structure of salinisporamine (**5**). With the X-ray structure of **5** in hand, it was possible to make a complete assignment of the NMR data listed in Table 2.

Salinorcinol (**3**) and salinisporamine (**5**) were tested for antimicrobial activity against *Bacillus subtilis* (UBC 344), *Staphylococcus aureus* (ATCC 43300), methicillin-resistant *S. aureus* (ATCC 33591), *Escherichia coli* (UBC 8161), *Pseudomonas aeruginosa* (ATCC 27853), and *Candida albicans* (ATCC 90028) using a standard disc diffusion assay. None of the compounds showed antimicrobial activity at a concentration of 40 µg/disc.

Given the range extension of *S. arenicola* RJA4486, the strains’ 16S rRNA gene sequence was utilized as a query in a BLASTN [11] search against the *Salinispora* genus. The results from that BLASTN search were utilized to construct a phylogenetic tree (Supporting Information). Additionally, the genomes of *S. arenicola* RJA4486 (5.6 Mbp, 140 contigs GenBank accession no. JAMQNB000000000) and *S. arenicola* RJA3005 (6.9 Mbp, 109 contigs GenBank accession no. JALPRT000000000) were sequenced utilizing a shotgun sequencing approach. The sequencing data from these two strains were analyzed for natural product biosynthetic gene clusters utilizing antiSMASH [12]. The results from antiSMASH were analyzed alongside data from the *S. arenicola* CNS-991 genome (GenBank Accession no. KB913036.1, collected from Fiji and available as a high-quality draft genome. Each putative gene cluster was analyzed for completeness by comparing the gene clusters to the MiBIG [13] analysis presented under the antiSMASH shell, along with performing BLASTP analysis for core biosynthetic genes. The results of this analysis demonstrate that all the analyzed *S. arenicola* strains contain complete gene clusters with 80% or higher sequence homology to characterized gene clusters for staurosporine, rifamycin, sporolide A, paramagnetoquinone, alkyl-O-dihydrogeranyl-methoxyhydroquinones, desferrioxamine, ketomemicin, and lymphostin. Additionally, gene clusters with shared sequence identity to calicheamicin (both at ~40%) and stenothricin (~30%) were found common to all analyzed strains. Nevertheless, despite belonging to the same species, each strain possesses a few biosynthetic gene clusters predicted to be unique (See Table 3 and Supplementary Materials), with RJA4486 containing the highest number of gene clusters predicted to belong to the terpene class. 

Due to the commonality of the rifamycin gene cluster to the natural products reported here, the rifamycin partial fragments found in *S. arenicola* RJA4486 and *S. arenicola* RJA3005 were aligned to the complete rifamycin gene cluster available in the sequenced genome of *S. arenicola* CNS-205, utilizing a MAUVE [14] alignment (Supplementary Materials). Results from the MAUVE alignment confirmed that the rifamycin gene cluster is found across multiple contigs in the sequenced genomes of *S. arenicola* RJA4486 and *S. arenicola* RJA3005; likely due to the draft genome status of each strain. At this stage, the gaps prevent detailed analysis for potential mutations.

## 3. Discussion

In this study, we have examined the unexplored metabolic potential of biosynthetically talented marine bacterial isolates through variation of culturing conditions and isolation of minor metabolites guided by chemical signatures such as UV chromatograms and unique NMR chemical shifts as an avenue to the discovery of new natural products. Our efforts have revealed the new polyketide natural products salinorcinol (**3**), salinacetamide (**4**), and salinisporamine (**5**). Our studies have revealed that wild-type *S. arenicola* strains from widely separated geographical regions and climatic zones produce unique natural products from the well-dispersed rifamycin gene cluster. Salinorcinol (**3**) and salinacetamide (**4**) were discovered as new natural products produced in culture by the *S. arenicola* strain RJA3005 isolated from sediments collected in the tropical southwestern Pacific waters off the coast of Papua New Guinea. Both **3** and **4** had been previously reported as metabolic products of laboratory mutated and/or engineered rifamycin-producing bacteria, but not as natural products. In particular, **3** was the product of a mutasynthetic study where an -AHBA mutant was fed various synthetically derived starting units [8]. The structural similarity of both compounds to a mutasynthesized compound (for **3**) [8] and an engineered compound (for **4**) [9] from a rifamycin producer strongly support that **3** and **4** are the products of the rifamycin gene cluster. Intriguingly, the 10,12-dihydroxy aromatic moiety of **3** would require the production of a unique starting unit as compared to the AHBA starting unit involved in rifamycin biogenesis [10]. The biogenesis of the necessary starting unit for **3** within the context of rifamycin biosynthesis is still unclear. In a related fashion, temperate ocean strain RJA4486 cultures produce the new putative rifamycin shunt product salinisporamine (**5**). When isolated with TFA, two sets of resonances were observed. One set of resonances is identical to that seen in the sample with no TFA present and the biggest difference is seen in the chemical shifts of H-13 that is adjacent to the 12-NH_2_. Likely when TFA is used under HPLC concentrations approximately half of the compound is protonated at 12-NH_2_ and charged and in the other half, it is not. Importantly, the isolation of *S*. *arenicola* strain RJA4486 from sediments collected in the temperate northeastern Pacific waters off the coast of B.C. appears to be a range extension for this species, which was thought to be confined to tropical habitats [4].

Despite the similar 16S rRNA gene sequences of *S. arenicola* strains RJA3005 and RJA4886, our work reveals chemodiverse compounds from these two strains of an identical species. This work underscores the importance of the sampling and screening process used for bioprospecting i.e., taxonomically identical strains should not be discarded, as these strains can produce strain-specific compounds [15,16]. This phenomenon can be explained by mobile biosynthetic gene clusters that are likely acquired by horizontal exchange among bacteria in an ecological niche to confer ecological fitness [17,18,19,20]. These acquired biosynthetic gene clusters can produce new chemical scaffolds or compounds, which can be exploited for medical or biochemical applications. The discovery of **3** and **4** as natural products provides an example of nature and human metabolic engineers using similar modifications of major pathways to sample biosynthetic chemical space. The work described herein reinforces the premise that exhaustive exploration of ‘biosynthetically talented’ bacterial strains is a productive way to find new natural products.

## 4. Materials and Methods

### 4.1. General Experimental Methods

Optical rotations were measured using a Jasco P-1010 Polarimeter with sodium light (589 nm). UV spectra were recorded with a Waters 2998 Photodiode Array Detector. ^1^H and ^13^C NMR spectra were recorded on a Bruker AV-600 spectrometer with a 5 mm CPTCI cryoprobe. ^1^H chemical shifts are referenced to the residual DMSO-*d_6_* (δ 2.49 ppm) and ^13^C chemical shifts are referenced to the DMSO-*d*_6_ solvent peak (δ 39.5 ppm). Low and high-resolution ESI-QIT-MS were recorded on a Bruker-Hewlett Packard 1100 Esquire–LC system mass spectrometer. Merck Type 5554 silica gel plates and Whatman MKC18F plates were used for analytical thin-layer chromatography. Reversed-phase HPLC purifications were performed on a Waters 1525 Binary HPLC Pump attached to a Waters 2998 Photodiode Array Detector. All solvents used for HPLC were Fisher HPLC grade.

### 4.2. Salinispora arenicola Strain RJA3005

#### 4.2.1. Bacterial Material and Isolation of **3** and **4**

*S. arenicola* strain RJA3005 was isolated from marine sediment collected in Papua New Guinea. Laboratory cultivation was carried out using nutrient-rich Marine Medium 1 (MM1- soluble starch: 10.0 g; yeast extract: 4.0 g; peptone: 18.0 g; sea water: 1.0 L; KBr: 0.001 g and FeSO_4_·7H_2_O: 0.0004 g. Solid media had agar added (2.0 g/L). *S. arenicola* strain RJA3005 was grown for 14 days on solid agar until a thick mat of orange leathery textured bacterial colonies had formed. The agar and mature mycelia were cut into small squares and immersed in EtOAc for extraction. The EtOAc soaked agar was filtered through paper to separate the agar from the supernatant (3×, ~2 L solvent per 8 L growth medium). The EtOAc portions were combined and the solvent removed in vacuo to give a thick red/brown oily-solid, with large portions only soluble in MeOH until further separation. The crude oily/solid was dissolved in a 9:1 mixture of H_2_O/MeOH prior to first partitioning between hexane/H_2_O and then CH_2_Cl_2_/H_2_O·MeOH was removed from the H_2_O layer *in vacuo* giving a deep golden aqueous solution containing reddish-brown oily-solids. EtOAc was added to completely dissolve the oily-solids and the EtOAc-soluble layer was removed and dried *in vacuo* giving a viscous oil that was the active fraction in the phosphatase inhibition assay. This material was chromatographed on Sephadex LH-20 (eluent: 4:1 MeOH/CH_2_Cl_2_) and fractions pooled by TLC similarities and bio-assayed. Active fractions were separated using step-gradient reversed-phase flash chromatography (H_2_O to MeOH), using C_18_ Sep-Paks. Fractions eluting in 4:1 H_2_O/MeOH and 3:2 H_2_O/MeOH (*v*/*v*), that contained ^1^H NMR peaks of interest, were further separated using semi-preparative C_18_ reversed-phase HPLC (InertSustain C18, 17:83 MeCN/H_2_O with 0.05% FA) to give pure samples of **3** and **4**.

#### 4.2.2. Salinorcinol (**3**)

Isolated as a white powder; [α]^25^_D_ −8.79° (c 3.5 g/100 mL, MeOH); ^1^H (DMSO-*d*_6_, 600 MHz) and ^13^C NMR (DMSO-*d*_6_, 150 MHz), see Table 2; HRESIMS [M + H]^+^ *m*/*z* 293.1046 (calcd for C_15_H_17_O_6_, 293.1025).

#### 4.2.3. Salinacetamide (**4**)

Isolated as a white powder; ^1^H (DMSO-*d_6_* 600 MHz) and ^13^C NMR (DMSO-*d_6_* 150 MHz), see Table 2; HRESIMS [M + H]^+^ *m*/*z* 334.1297 (calcd for C_17_H_20_O_6_N, 334.1291).

### 4.3. Salinispora arenicola Strain RJA4486

#### 4.3.1. Bacterial Material

*S. arenicola* RJA4486, was isolated on Marine Medium 10 (10.0 g of glucose, 4.0 g of yeast extract, 2.0 g of peptone, 100 ug/mL of cyclohexamide, 5 ug/mL of rifampicin, 15.0 g of agar, 1 L of seawater) from marine sediment collected at Barkley Sound, British Columbia, Canada at a depth of 82 m (N 48°52.830′, W 125°09.838′). NCBI blast analysis of the partial 16S rRNA sequence of *Salinispora Arenicola* isolate RJA4486 (deposited in GenBank with an accession number OM721757) is 100% identical to *Salinispora arenicola* ATCC BAA-917 strain CNH-643 (NR_042725.1) isolated from the coarse sand of the Bahamas [16].

#### 4.3.2. Isolation of Salinisporamine (**5**)

Strain RJA4486 was cultured on 70 trays of solid agar, equivalent to 22.0 L volume of the marine medium 1 (MM1) (100.0 g of soluble starch, 40.0 g of yeast extract, 20.0 g of peptone, 0.01 g of FeSO_4_·7H_2_O, 0.01 g of KBr, 180.0 g agar, 10 L seawater) at RT for 14 days. The mature cultures were sliced into small squares containing the *S. arenicola* biomass and the media and extracted twice with EtOAc. The combined EtOAc extracts were concentrated *in vacuo* and partitioned between H_2_O (750 mL) and EtOAc (3 × 200 mL). The EtOAc extracts were combined and concentrated in vacuo and the EtOAc soluble material was chromatographed on Sephadex LH20 with 4:1 MeOH/CH_2_Cl_2_ as eluent. From two earlier eluting fractions rifamycin W (**1**) [6] and staurosporine (**2**) [7] were isolated. A later eluting fraction was subjected to Si gel flash chromatography (step gradient: 19:1 hexanes/EtOAc to EtOAc to 1:9 MeOH/EtOAc and to MeOH, 2 g Sep pak). The 1:1 hexanes/EtOAc fraction was further fractionated by C-18 reversed-phase HPLC using an InertSustain, 5 μm, 25 × 1 cm column, with 7:3 (0.05% TFA/H_2_O)/MeCN as eluent to give the TFA salt of salinisporamine (**5**) which was then desalted on C-18 reversed-phase HPLC using the same column but with 7:3 H_2_O/MeCN as eluent to yield 0.4 mg of salinisporamine (**5**).

#### 4.3.3. Salinisporamine (**5**)

Isolated as orange blade shaped crystals; mp at 235 °C appeared to decompose; UV [7:3 H_2_O)/MeCN] λ_max_ 195, 217, 282, 322 nm; ^1^H NMR see Table 3; ^13^C NMR, see Table 3; positive ion HRESIMS [M + H]^+^ *m*/*z* 326.1022 (calcd for C_18_H_16_NO_5_, 326.1028).

### 4.4. Genome Extraction and Sequencing

Genomic DNA of S. arenicola RJA 3005 and S. arenicola RJA 4486 were prepared using a salting out procedure [21]. In brief, 20 μL of a spore solution of strains were added to 50 mL of MM1 medium. After incubation for 8 days at 30 °C with shaking at 200 rpm in a 250 mL flask equipped with sterile beads, the mycelia were collected by centrifugation at 4000 rpm from 5 mL of cell suspension. Pellets were resuspended in 1 mL of STE buffer (75 mM NaCl, 25 mM EDTA pH 8.0, 20 mM Tris-HCl pH 7.5), then a lysozyme solution was added to 0.3 mg/mL. After lysis at 30 °C for 15 min, 0.1 mL of a 10% (*w*/*v*) SDS solution was added and then mixed slowly by inversion and incubated for 10 min at 55 °C. NaCl was added to 1.25 M and mixed thoroughly by inversion before one equivalent chloroform was added to precipitate proteins. The two-phase solution was mixed by inversion for 30 min at room temperature and centrifuged for 20 min at 6000 rpm. The upper aqueous phase was transferred to a new tube and 0.6 (*v*/*v*) equivalent isopropanol was added for salting out of DNA. The liquid was removed and 70% (*v*/*v*) of ethanol was added to wash the DNA before dissolving the resulting pellet in TE buffer (pH 8.0). Sequencing was performed at the Chinese National Human Genome Center (Shanghai, China). A total of 1 μg of genomic DNA was fragmented by sonication and ~500 bp and ~300 bp fragments were recovered by agarose gel electrophoresis to construct libraries using TruSeqTM DNA Sample Prep Kit—Set A (Illumina, USA). The libraries were then amplified by TruSeq PE Cluster Kit (Illumina, USA) and two libraries were sequenced on the Illumina Hiseq2000. The 300 bp and 500 bp libraries yielded 0.99 Gbp and 0.7 Gbp, respectively for S. arenicola RJA 3005 and 1.19 Gbp and 0.73 Gbp, respectively for S. arenicola RJA 4486. After sequence assembly using Velvet 1.2.03 [22], the final assembly consisted of 6.9 Mbp of non-redundant sequence across 109 contigs (coverage is ~383×) for S. arenicola RJA 3005 and 5.6 Mbp of non-redundant sequence across 140 contigs for S. arenicola RJA 4486. Gene analysis and functional annotation were performed using Glimmer 3.02 [23], combined with 2ndFind (http://biosyn.nih.go.jp/2ndfind/ accessed on 10 February 2019) and BlastP [24].

### 4.5. Bioinformatic Analysis

Bioinformatic programs were used at default settings. Fasta files produced during sequencing were uploaded to antiSMASH. All gene clusters predicted from antiSMASH were then visually inspected for complete gene clusters or truncated gene clusters. Gene clusters were cross-referenced with MiBIG available in the antiSMASH shell. Core genes and key accessory genes were utilized for further BLASTP searches before assigning a natural product class and closest homologous gene cluster. Mauve alignments were completed utilizing the *S. arenicola* CNS-205 rifamycin gene cluster extracted from antiSMASH. The contigs containing partial rifamycin gene clusters of *S. arenicola* RJA 4486 and RJA 3005 were reorganized in alignment with the complete gene cluster using the Mauve, ‘Move Contigs’ tool. The list of genes available from antiSMASH analysis and the genome sequencing was then aligned to confirm the incomplete sequencing status of the rifamycin gene cluster in *S. arenicola* RJA4486 and RJA3005.

## Figures and Tables

**Table 1:** NMR data for salinorcinol (**3**) and salinacetamide (**4**) recorded in DMSO-*d_6_* at 600 MHz.

	Salinorcinol (3)		Salinacetamide (4)		
**Position**		δ_C_/δ_N_	δ_H_ (*J* = Hz)	δ_C_/δ_N_	δ_H_ (*J* = Hz)
**1**	C	165.2	-	165.2	-
**2**	C	96.6	-	96.8	-
**3**	C	165.2	-	164.7	-
**4**	CH	100.0	5.99 s	100.7	5.99 s
**5**	C	164.2	-	164.1	-
**6**	CH	45.4	2.64 q (~7.1)	46.6	2.66 q (~7.1)
**7**	CH	74.7	4.36 dd (4.0, 8.5)	74.8	4.42 dd (4.0, 8.5)
**8**	C	145.4	-	145.1	-
**9**	CH	104.8	6.16 d (2.2)	109.3	6.39 br. s
**10**	C	157.9	-	157.0	-
**11**	CH	101.5	6.09 t (2.2)	106.1	7.14 br.t (1.68)
**12**	C	157.9	-	168.3	-
**13**	CH	104.8	6.16 d (2.2)	108.5	6.89 br.s
**14**	CH_3_	8.5	1.75 s	9.02	1.77 s
**15**	CH_3_	14.9	0.85 d (7.1)	14.9	0.84 d (7.1)
**16**	HNCOCH_3_	-	-	140.0	-
**17**	HNCOCH_3_	-	-	24.2	2.01 s
**12-OH**	OH	-	9.15	-	-
**12′-NH**	NH	-	-	−247.1	9.80
**10-OH**	OH	-	9.15	-	9.33
**7-OH**	OH	-	5.32 d (4.0)	-	5.42 d (4.0)
**3-OH**	OH	-	11.13	-	11.07

The underlined character is the carbon associated with the chemical shift within the acetamide functional group.

**Table 2:** NMR data for salinisporamine (**5**) recorded in DMSO-*d*_6_ at 600 MHz.

Salinisporamine (5)		
**Position**	^13^C/^15^N (δ)	^1^H (δ, multiplicity (*J* Hz))
**1**	163.6	-
**2**	121.9	-
**3**	144.2	7.30, q (1.0)
**4**	112.0	-
**5**	152.1	-
**6**	117.7	-
**7**	159.4 br	-
**8**	129.5	-
**9**	130.5	7.89, q (0.6)
**10**	123.2	-
**11**	180.8	-
**12**	149.5	-
**13**	102.6	5.58, s
**14**	181.8	-
**15**	131.4	-
**16**	16.2	2.02, d (1.0)
**17**	14.7	1.59, s
**18**	16.7	2.30, bs
**7-OH**	-	10.10, s
**12-NH**	no	7.05, bs (half height 232.2 Hz)

The underlined character is the atoms with described chemical shifts for the displayed functional group.

**Table 3:** Closest Homologous NRP, PKS, and Hybrid Gene Clusters for *S. arenicola* Strains.

	*S. arenicola* CNS-991 KB913036.1	*S. arenicola* RJA3005	*S. arenicola* RJA4486
**NRP**	Vazabitide A	Thiocoralin	Nematophin
Closest Homologous Gene Cluster	Stenothricin	Stenothricin	Stenothricin
		Truncated	Myxochelin A
		Tallysomycin	Tallysomycin
			Unknown
**Hybrid**	Leinamycin	Maduropeptin	Thalassospiramide A
Closest Homologous Gene Cluster	Lymphostin	Ikarugumycin	Lymphostin
	Calicheamicin × 2	Calicheamicin × 2	Calicheamicin × 2
	Polyoxypeptin	Neocazinostatin	
	Collismycin		
**PKS**	Rifamycin *	PKS Rifamycin *	PKS Rifamycin *
Closest Homologous Gene Cluster	Sporolide A	Sporolide A	Sporolide A and B
	Kedarcidin	Mediamycin	Naphthyridinomycin
	Paramagnetoquinone	Paramagnetoquinone	Paramagnetoquinone
	Alkyl-O-dihydrogeranyl-methoxyhydroquinones	Alkyl-O-dihydrogeranyl-methoxyhydroquinones	Alkyl-O-dihydrogeranyl-methoxyhydroquinones
	Herboxidiene		Amycomicin

* The rifamycin gene cluster was found over: green, two contigs; red, four contigs; brown, five contigs.

## Data Availability

NMR FIDs are available from R.J.A.

## Supplementary Materials

The following supporting information can be downloaded at: https://www.mdpi.com/article/10.3390/molecules27113569/s1, 1D and 2D NMR spectra for compounds **3**, **4,** and **5**; Experimental details for X-ray diffraction analysis of **5** and additional secondary metabolite gene cluster details.

## Sample Availability

Samples of the compounds are not available from the authors.

## References

[1] Jensen P.R., Moore B.S., Fenical W. (2015). The marine actinomycete genus Salinispora: A model organism for secondary metabolite discovery. Nat. Prod. Rep..

[2] Fenical W., Jensen P.R. (2006). Developing a new resource for drug discovery: Marine actinomycete bacteria. Nat. Chem. Biol..

[3] Pan R., Bai X., Chen J., Zhang H., Wang H. (2019). Exploring Structural Diversity of Microbe Secondary Metabolites Using OSMAC Strategy: A Literature Review. Front. Microbiol..

[4] Jensen P.R., Mafnas C. (2006). Biogeography of the marine actinomycete Salinispora. Environ. Microbiol..

[5] Bauermeister A., Velasco-Alzate K., Dias T., Macedo H., Ferreira E.G., Jimenez P.C., Lotufo T.M.C., Lopes N.P., Gaudêncio S.P., Costa-Lotufo L.V. (2018). Metabolomic Fingerprinting of *Salinispora* from Atlantic Oceanic Islands. Front. Microbiol..

[6] Martinelli E., Gallo G.G., Antonini P., White R.J. (1974). Structure of rifamycin W, a novel ansamycin from a mutant of *Norcardia mediterranei*. Tetrahedron.

[7] Omura S., Iwai Y., Hirano A., Nakagawa A., Awaya J., Tsuchiya H., Takahashi Y., Masuma R. (1977). New alkaloid AM-2282 of *Streptomyces* origin: Taxonomy, fermentation, isolation and preliminary characterization. J. Antibiot..

[8] Bułyszko I., Dräger G., Klenge A., Kirschning A. (2015). Evaluation of the Synthetic Potential of an AHBA Knockout Mutant of the Rifamycin Producer *Amycolatopsis mediterranei*. Chem. Eur. J..

[9] Xu J., Wan E., Kim C.-J., Floss H.G., Mahmud T. (2005). Identification of tailoring genes involved in the modification of the polyketide backbone of rifamycin B by *Amycolatopsis mediterranei* S699. Microbiology.

[10] August P.R., Tang L., Yoon Y.J., Ning S., Müller R., Yu T.-W., Taylor M., Hoffmann D., Kim C.-G., Zhang X. (1998). Biosynthesis of ansmycin antibiotic rifamycin: Deductions from the molecular analysis of the rif biosynthetic gene cluster of *Amycolatopsis mediterranei* S699. Chem. Biol..

[11] Altschul S.F., Gish W., Miller W., Myers E.W., Lipman D.J. (1990). Basic Local Alignment Search Tool. J. Mol. Biol..

[12] Blin K., Wolf T., Chevrette M.G., Lu X., Schwalen C.J., Kautsar S.A., Suarez Duran H.G., de Los Santos E.L.C., Kim H.U., Nave M. (2017). AntiSMASH 4.0-Improvements in Chemistry Prediction and Gene Cluster Boundary Identification. Nucl. Acids Res..

[13] Kautsar S.A., Blin K., Shaw S., Navarro-Muñoz J.C., Terlouw B.R., van der Hooft J.J.J., van Santen J.A., Tracanna V., Suarez Duran H.G., Pascal Andreu V. (2019). MIBiG 2.0: A repository for biosynthetic gene clusters of known function. Nucl. Acids Res..

[14] Darling A.C.E., Mau B., Blattner F.R., Perna N.T. (2004). Mauve: Multiple Alignment of Conserved Genomic Sequence With Rearrangements. Genome Res..

[15] Sabido E.M., Tenebro C.P., Trono D.J.V.L., Vicera C.V.B., Leonida S.F.L., Maybay J.J.W.B., Reyes-Salarda R., Amago D.S., Aguadera A.M.V., Octaviano M.C. (2021). Insights into the Variation in Bioactivities of Closely Related *Streptomyces* Strains from Marine Sediments of the Visayan Sea against ESKAPE and Ovarian Cancer. Mar. Drugs.

[16] Tenebro C.P., Trono D., Vicera C., Sabido E.M., Ysulat J.A., Macaspac A., Tampus K.A., Fabrigar T., Saludes J.P., Dalisay D.S. (2021). Multiple strain analysis of *Streptomyces* species from Philippine marine sediments reveals intraspecies heterogeneity in antibiotic activities. Sci. Rep..

[17] Egan S., Wiener P., Kallifidas D., Wellington E.M. (1998). Transfer of streptomycin biosynthesis gene clusters within Streptomycetes isolated from soil. Appl. Environ. Microbiol..

[18] Ziemert N., Lechner A., Wietz M., Millán-Aguiñaga N., Chavarria K.L., Jensen P.R. (2014). Diversity and evolution of secondary metabolism in the marine actinomycete genus *Salinispora*. Proc. Natl. Acad. Sci. USA.

[19] Letzel A.C., Li J., Amos G., Millán-Aguiñaga N., Ginigini J., Abdelmohsen U.R., Gaudêncio S.P., Ziemert N., Moore B.S., Jensen P.R. (2017). Genomic insights into specialized metabolism in the marine actinomycete *Salinispora*. Environ. Microbiol..

[20] Maldonado L.A., Fenical W., Jensen P.R., Kauffman C.A., Mincer T.J., Ward A.C., Bull A.T., Goodfellow M. (2005). Salinispora arenicola gen. nov., sp. nov. and Salinispora tropica sp. nov., obligate marine actinomycetes belonging to the family Micromonosporaceae. Int. J. Syst. Evol. Microbiol..

[21] Kieser T., Bibb M.J., Buttner M.J., Chater K.F., Hopwood D.A. (2000). Practical Streptomyces Genetics.

[22] Zerbino D.R., Birney E. (2008). Velvet: Algorithms for de novo short read assembly using de Bruijn graphs. Genome Res..

[23] Delcher A.L., Harmon D., Kasif S., White O., Salzberg S.L. (1999). Improved microbial gene identification with GLIMMER. Nucl. Acids Res..

[24] McGinnis S., Madden T.L. (2004). BLAST: At the core of a powerful and diverse set of sequence analysis tools. Nucl. Acids Res..

