# Supplementary material for: Natural Products Produced in Culture by Biosynthetically Talented Salinispora arenicola Strains Isolated from Northeastern and South Pacific Marine Sediments
Source: Molecules. 2022 Jun 2;27(11):3569. doi: 10.3390/molecules27113569 (PMC9181873; doi:10.3390/molecules27113569)
Supplement: Supplementary file 1 [file molecules-27-03569-s001.zip › molecules-1724457-supplementary.pdf]

Supplementary Materials

Natural Products Produced in Culture by Biosynthetically Talented *Salinispora arenicola* Strains Isolated from Northeastern and South Pacific Marine Sediments

David E. Williams [1,†], Kalindi D. Morgan [1,2,3,†], Doralyn S. Dalisay [4], Teatulohi Matainaho [5], Elodie Perrachon [1], Noemie Viller [1], Maïlys Delcroix [1], Jeanne Gauchot [1], Haruka Niikura [2], Brian O. Patrick [2], Katherine S. Ryan [2,*] and Raymond J. Andersen [1,*]

1 Departments of Chemistry and Earth, Ocean and Atmospheric Sciences, University of British Columbia, Vancouver, BC V6T 1Z1, Canada; davewill@chem.ubc.ca (D.E.W.); elodie.perrachon@ensiacet.fr (E.P.); noemie.viller@ensiacet.fr (N.V.); mailys.delcroix@ensiacet.fr (M.D.); jeanne.gauchot@ensiacet.fr (J.G.)
2 Department of Chemistry, University of British Columbia, Vancouver, BC V6T 1Z1, Canada; harukaniikura13826@gmail.com (H.N.); bpatrick@chem.ubc.ca (B.O.P.); ksryan@chem.ubc.ca (K.S.R.)
3 Department of Chemistry and Biochemistry, University of Northern British Columbia, Prince George, BC V2N 4Z9, Canada; kalindi.morgan@unbc.ca (K.D.M.)
4 Center for Chemical Biology and Biotechnology (C2B2), Department of Biology, College of Liberal Arts, Sciences and Education, University of San Agustin, Iloilo, 5000, Philippines: ddalisay@usa.edu.ph
5 School of Medicine and Health Sciences, University of Papua New Guinea, P.O. Box 5623, Boroko, NCD, Papua New Guinea; lmatainaho@yahoo.com
* Correspondence: raymond.andersen@ubc.ca
† These authors contributed equally to this work.

Table of contents

Page SI-3: ^{1}H NMR Spectrum of Salinorcinol (**3**) recorded at 600 MHz in DMSO-d_6.
Page SI-4: Expanded ^{1}H NMR Spectrum of Salinorcinol (**3**) with peak picking recorded at 600 MHz in DMSO-d_6.
Page SI-5: Expanded ^{1}H NMR Spectrum of Salinorcinol (**3**) with peak picking recorded at 600 MHz in DMSO-d_6.
Page SI-6: ^{13}C NMR Spectrum of Salinorcinol (**3**) recorded at 150 MHz in DMSO-d_6.
Page SI-7: gradCOSY60 NMR Spectrum of Salinorcinol (**3**) recorded at 600 MHz in DMSO-d_6.
Page SI-8: gradHSQC NMR Spectrum of Salinorcinol (**3**) recorded at 600 MHz in DMSO-d_6.
Page SI-9: gradHMBC NMR Spectrum of Salinorcinol (**3**) recorded at 600 MHz in DMSO-d_6.
Page SI-10: tROESY NMR Spectrum of Salinorcinol (**3**) recorded at 600 MHz in DMSO-d_6.
Page SI-11: ^{1}H NMR Spectrum of for Salinacetamide (**4**) recorded at 600 MHz in DMSO-d_6.
Page SI-12: Expanded ^{1}H NMR Spectrum of Salinacetamide (**4**) with peak picking recorded at 600 MHz in DMSO-d_6.
Page SI-13: Expanded ^{1}H NMR Spectrum of Salinacetamide (**4**) with peak picking recorded at 600 MHz in DMSO-d_6.
Page SI-14: ^{13}C NMR Spectrum of Salinacetamide (**4**) recorded at 150 MHz in DMSO-d_6.
Page SI-15: gradCOSY60 NMR Spectrum of Salinacetamide (**4**) recorded at 600 MHz in DMSO-d_6.
Page SI-16: gradHSQC NMR Spectrum of Salinacetamide (**4**) recorded at 600 MHz in DMSO-d_6.
Page SI-17: gradHMBC NMR Spectrum of Salinacetamide (**4**) recorded at 600 MHz in DMSO-d_6.
Page SI-18: tROESY NMR Spectrum of Salinacetamide (**4**) recorded at 600 MHz in DMSO-d_6.
Page SI-19: grad^{15}N-HSQC NMR Spectrum of Salinacetamide (**4**) recorded at 600 MHz in DMSO-d_6.

Page SI-20: ^{1}H NMR Spectrum of Salinisporamine (**5**) in the presence of TFA recorded at 600 MHz in DMSO-d_6.
Page SI-21: ^{1}H NMR Spectrum of Salinisporamine (**5**) recorded at 600 MHz in DMSO-d_6.
Page SI-22: ^{13}C NMR Spectrum of Salinisporamine (**5**) recorded at 150 MHz in DMSO-d_6.
Page SI-23: gradCOSY60 NMR Spectrum of Salinisporamine (**5**) recorded at 600 MHz in DMSO-d_6.
Page SI-24: gradHSQC NMR Spectrum of Salinisporamine (**5**) recorded at 600 MHz in DMSO-d_6.
Page SI-25: gradHMBC NMR Spectrum of Salinisporamine (**5**) recorded at 600 MHz in DMSO-d_6.
Page SI-26: tROESY NMR Spectrum of Salinisporamine (**5**) recorded at 600 MHz in DMSO-d_6.
Page SI-27 to SI-32: Details of the X-ray Diffraction Analysis of Salinisporamine (**5**).
Page SI-33: Phylogenetic Tree of the *Salinispora* Genus with RJA 4486 and RJA 3005.
Page SI-34: Chart Comparing Biosynthetic Gene Clusters Classes of RJA 4486 and RJA 3005.
Page SI-35: MAUVE Alignments between *S. arenicola* CNS-205 rifamycin gene cluster with RJA 4486 and RJA 3005.

^{1}H NMR Spectrum of Salinorcinol (**3**) recorded at 600 MHz in DMSO-d_6

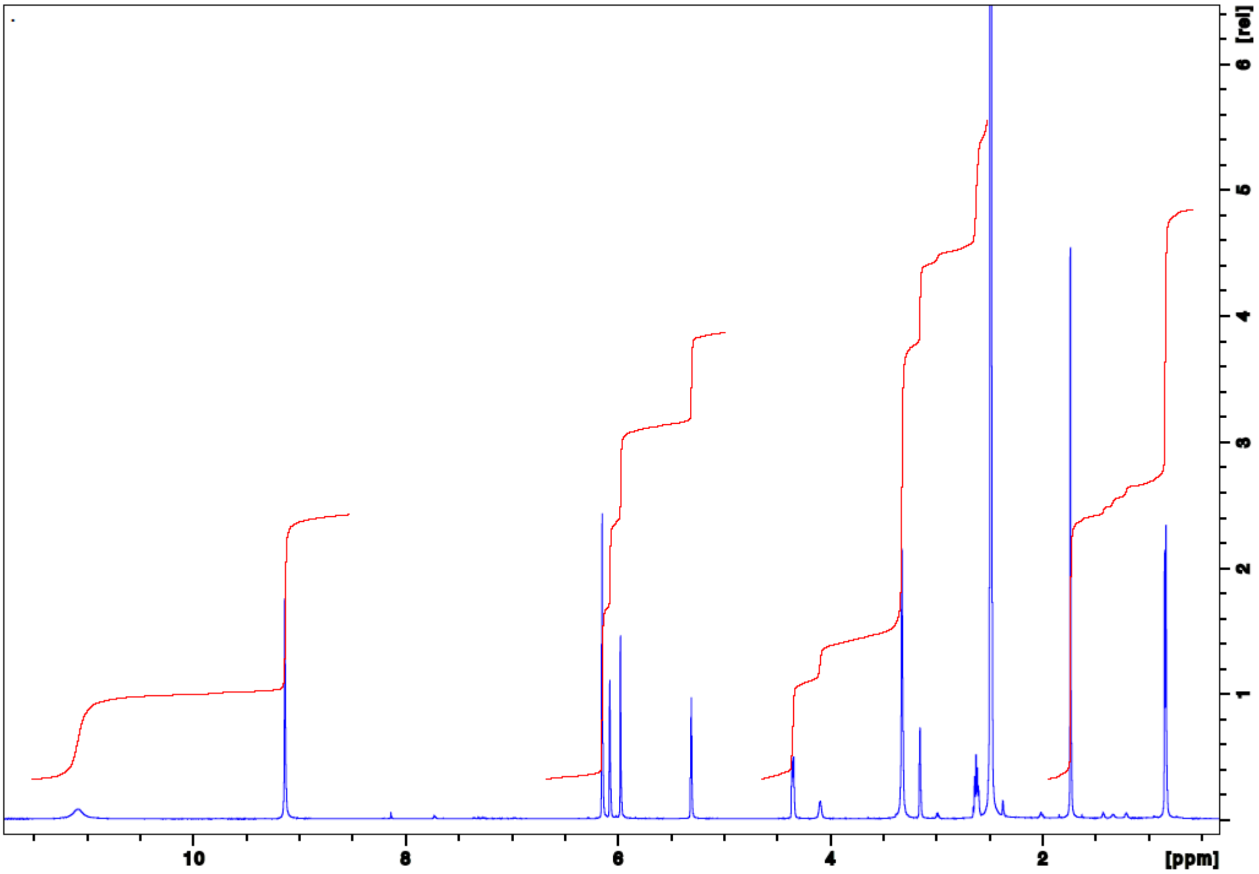

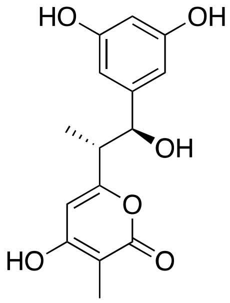

3

Expanded ^1H NMR Spectrum of Salinorcinol (**3**) with peak picking recorded at 600 MHz in DMSO-d_6

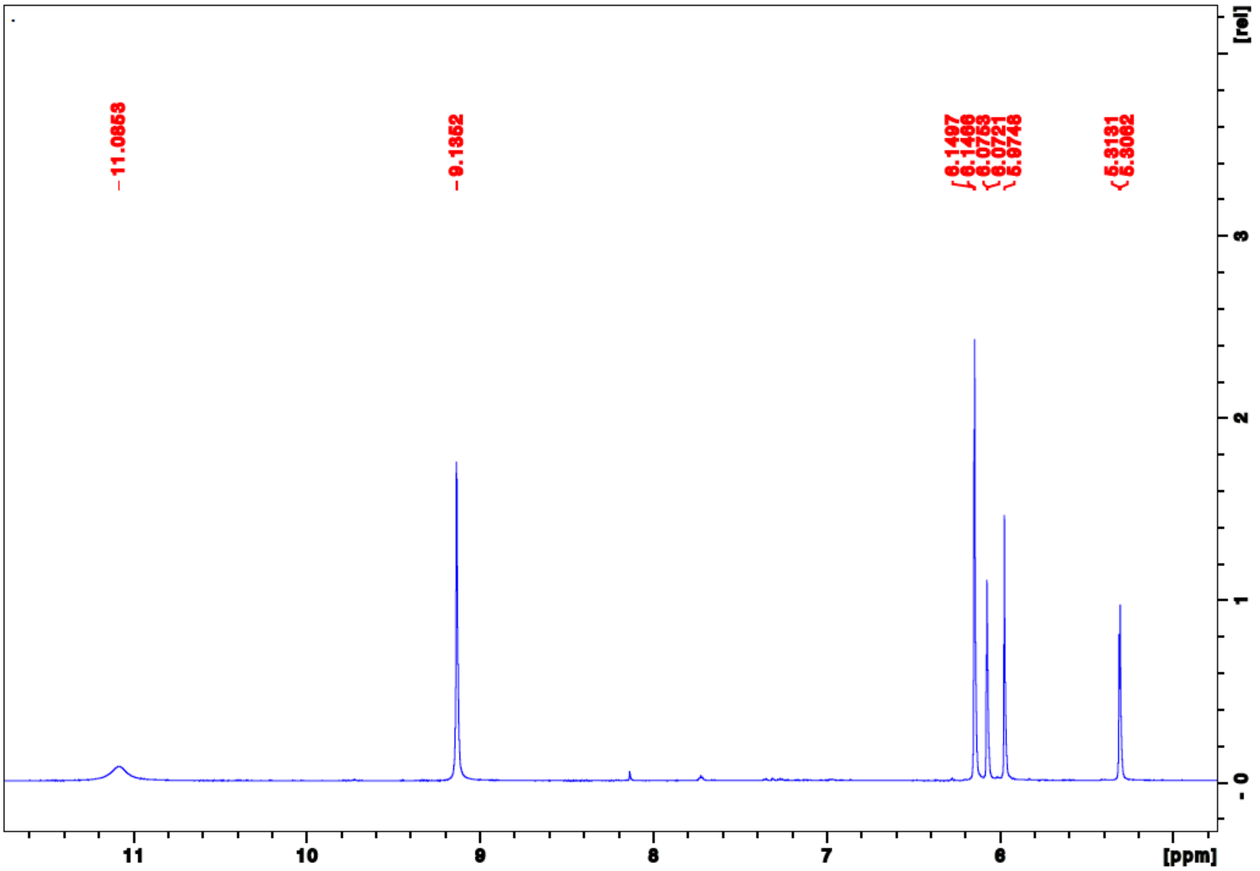

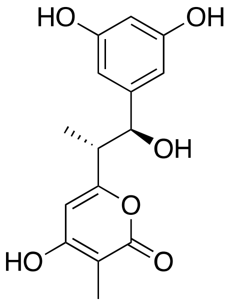

3

Expanded ^{1}H NMR Spectrum of Salinorcinol (**3**) with peak picking recorded at 600 MHz in DMSO-d_6

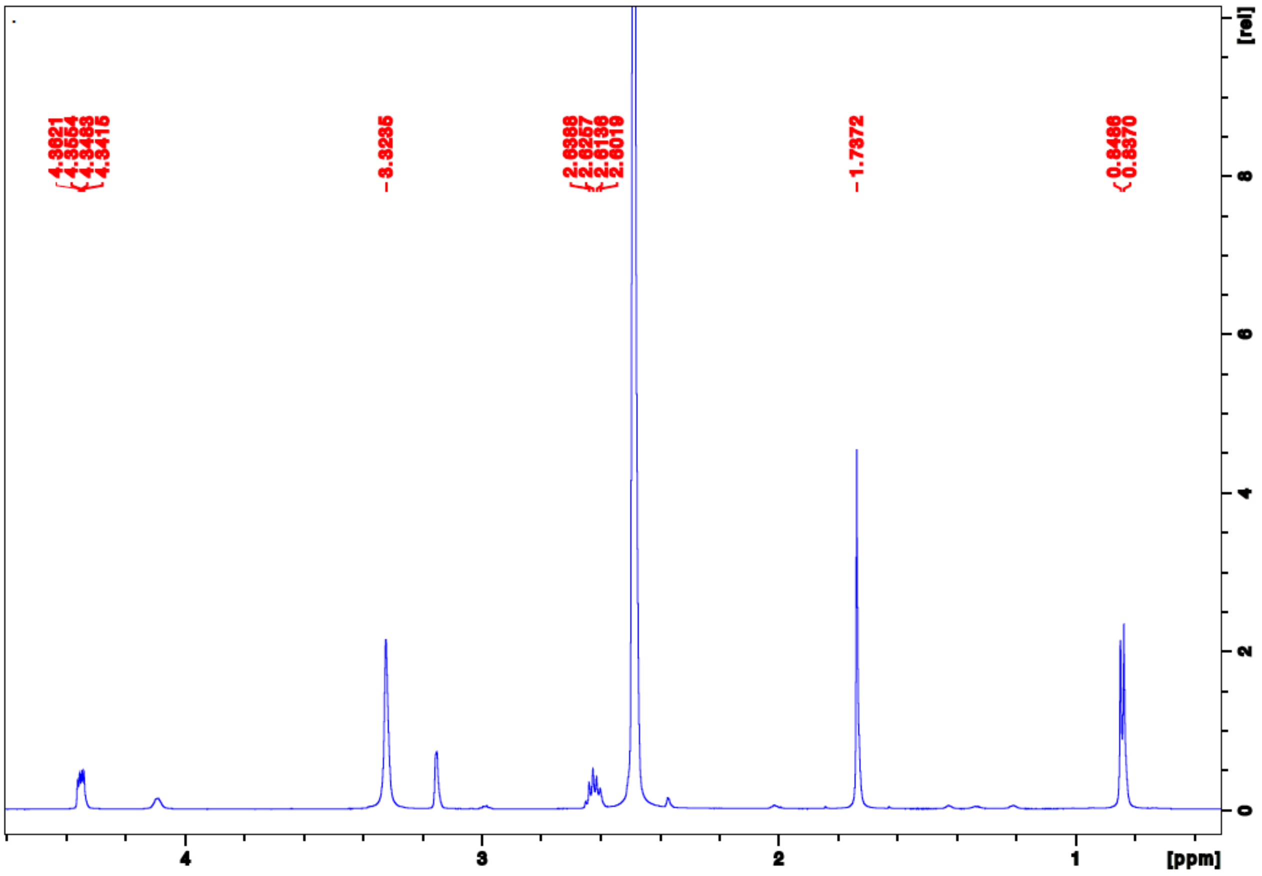

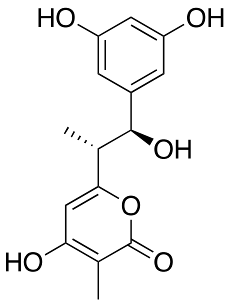

3

^{13}C NMR Spectrum of Salinorcinol (**3**) recorded at 150 MHz in DMSO-d_6

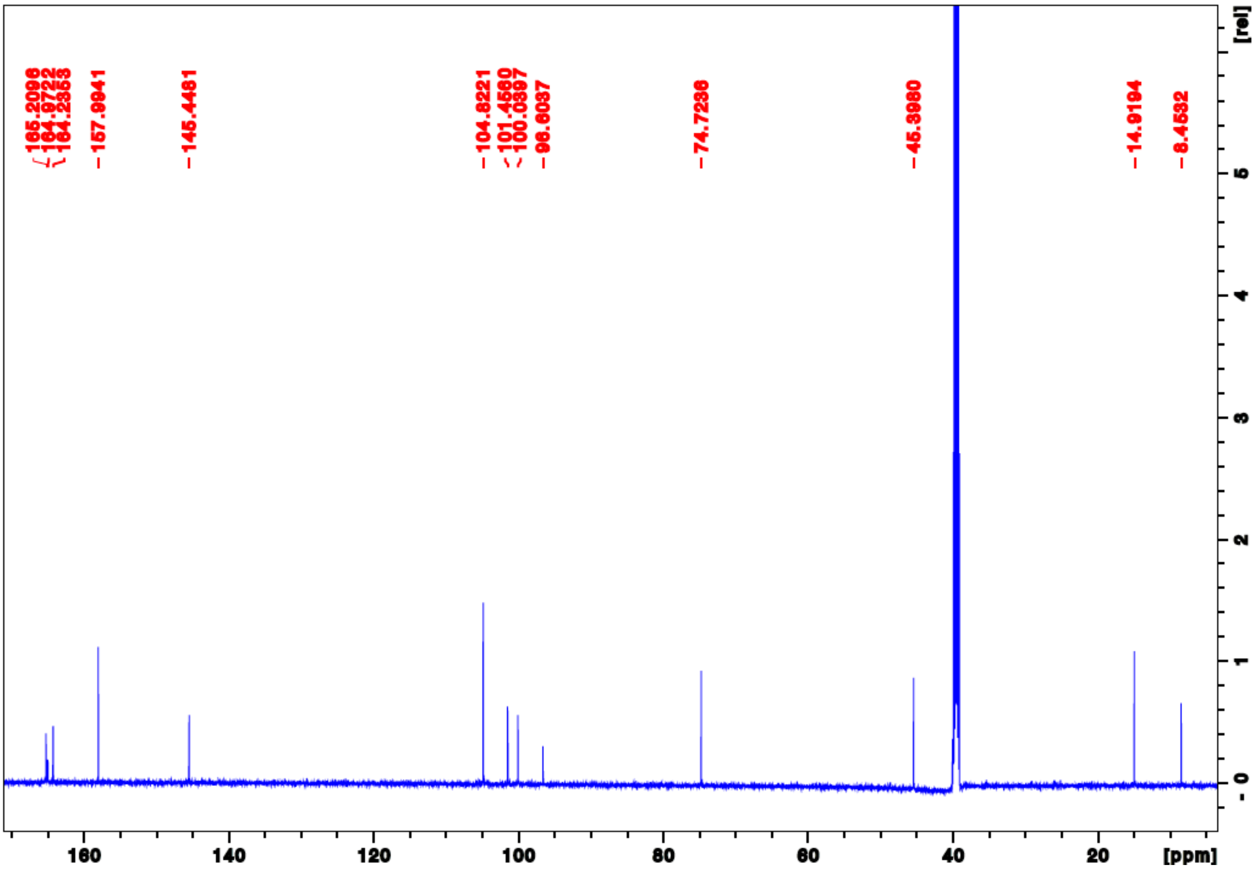

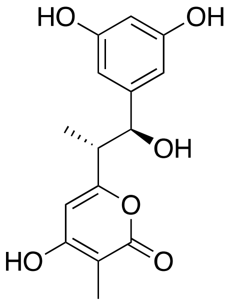

3

gradCOSY60 NMR Spectrum of Salinorcinol (**3**) recorded at 600 MHz in DMSO-d_6

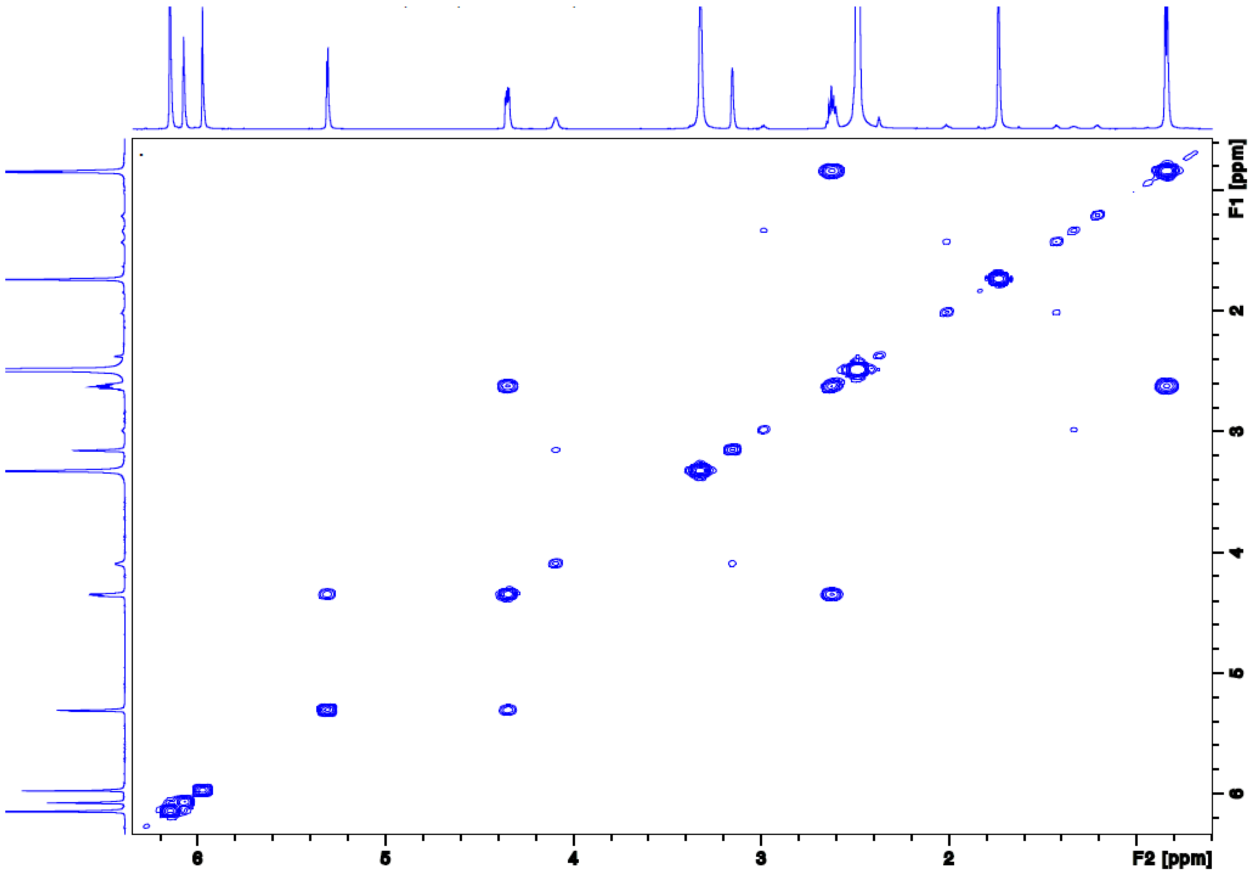

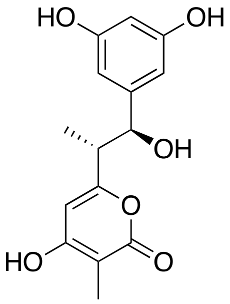

3

gradHSQC NMR Spectrum of Salinorcinol (**3**) recorded at 600 MHz in DMSO-d_6

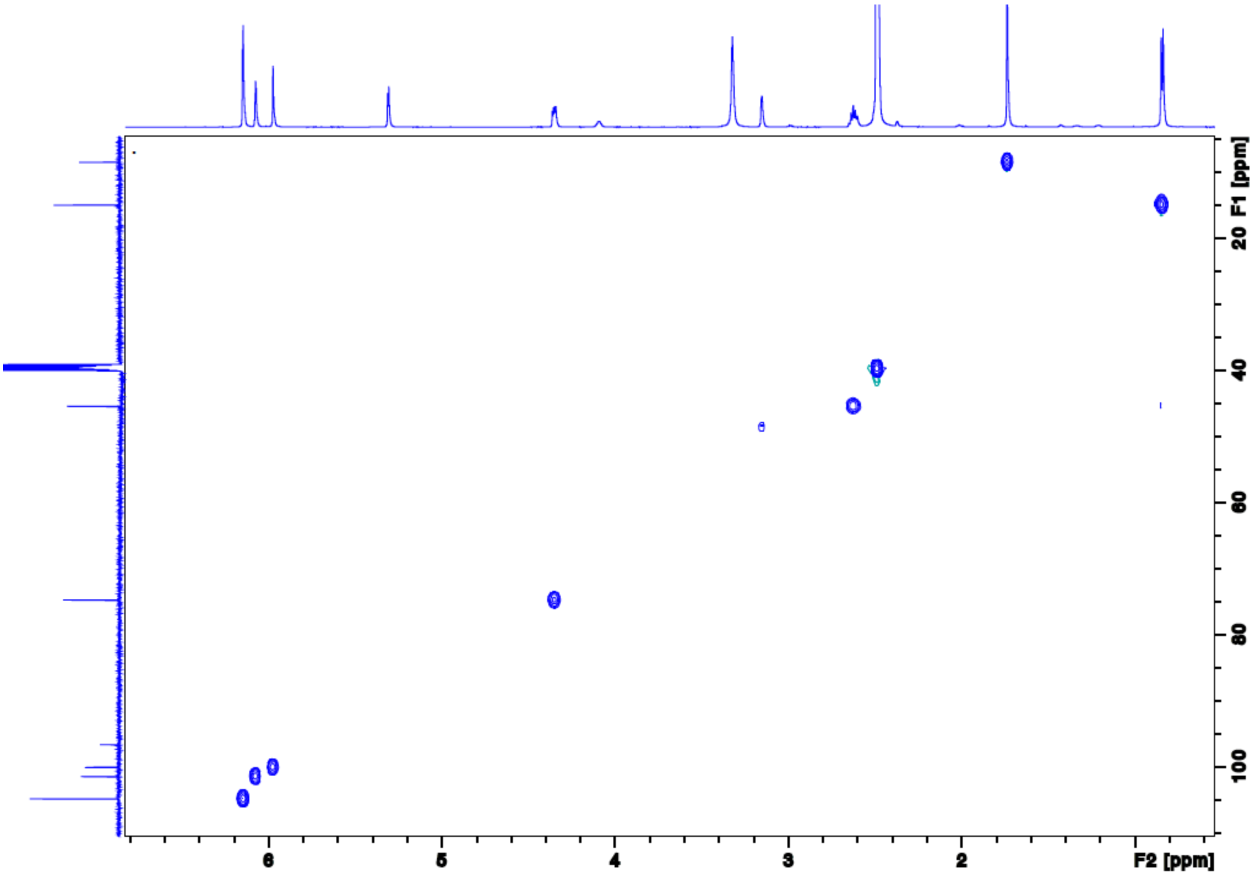

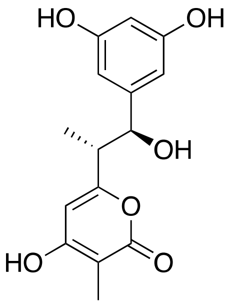

3

gradHMBC NMR Spectrum of Salinorcinol (**3**) recorded at 600 MHz in DMSO-d_6

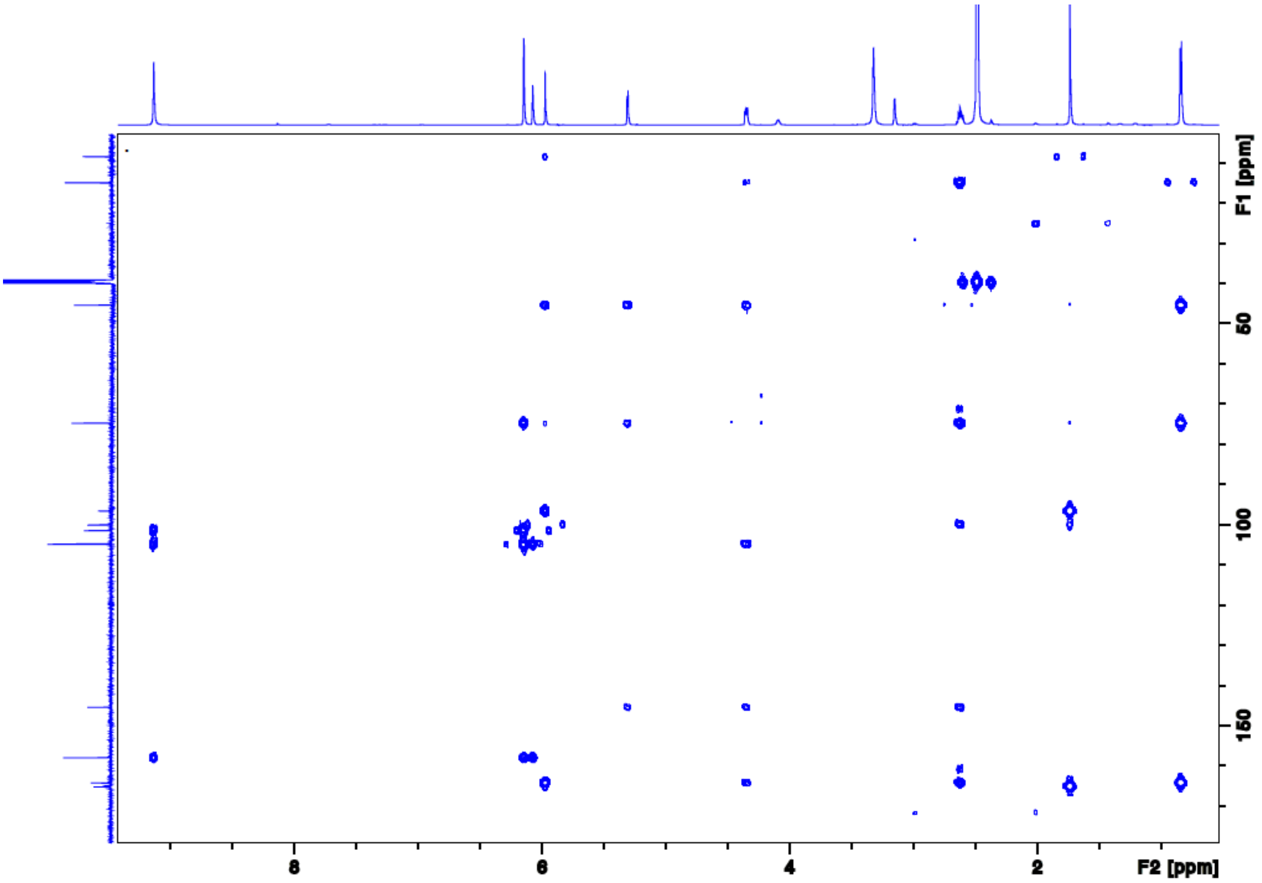

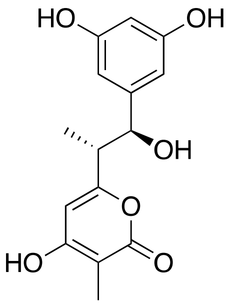

3

tROESY NMR Spectrum of Salinorcinol (**3**) recorded at 600 MHz in DMSO-d_6

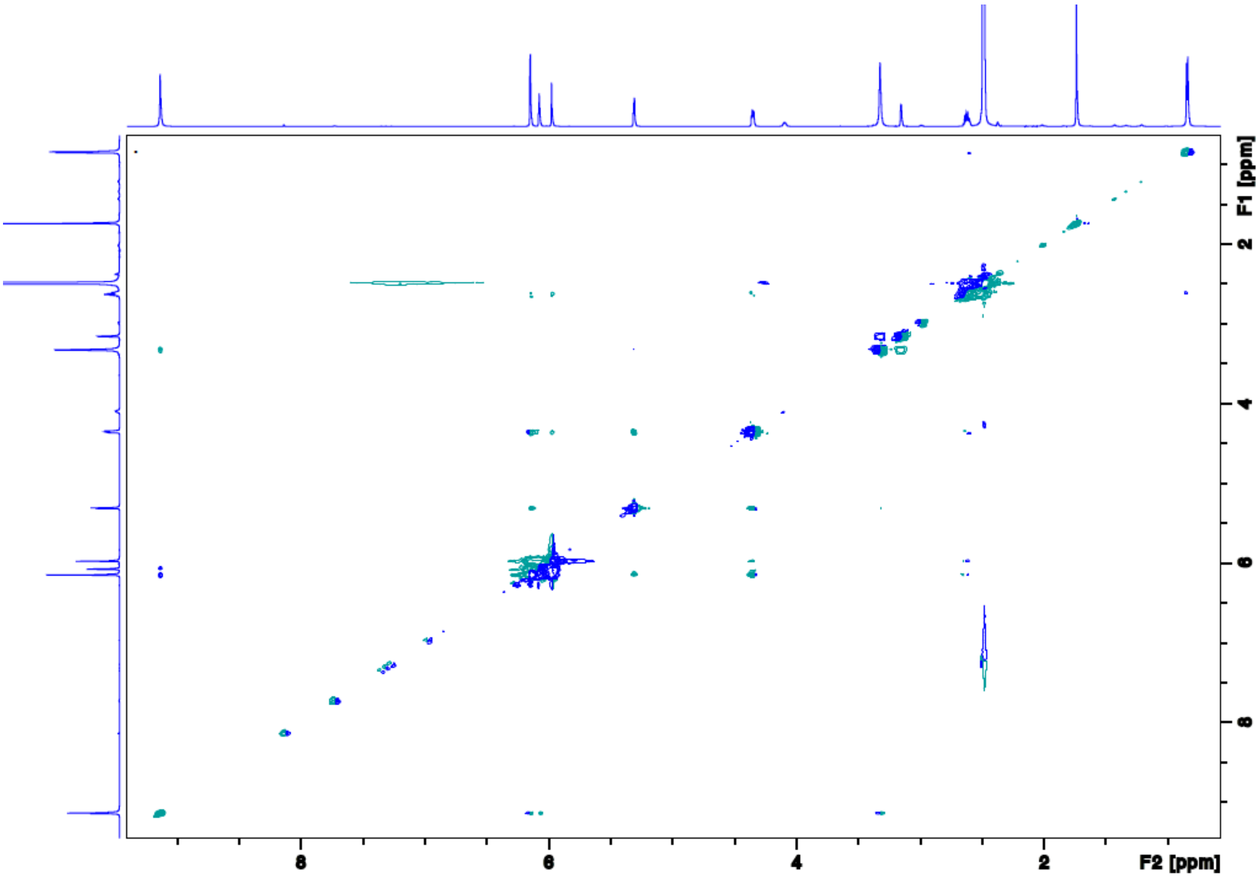

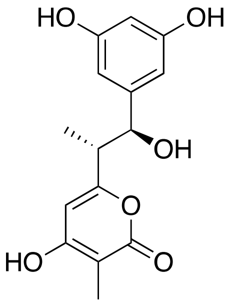

3

^{1}H NMR Spectrum of for Salinacetamide (**4**) recorded at 600 MHz in DMSO-d_6

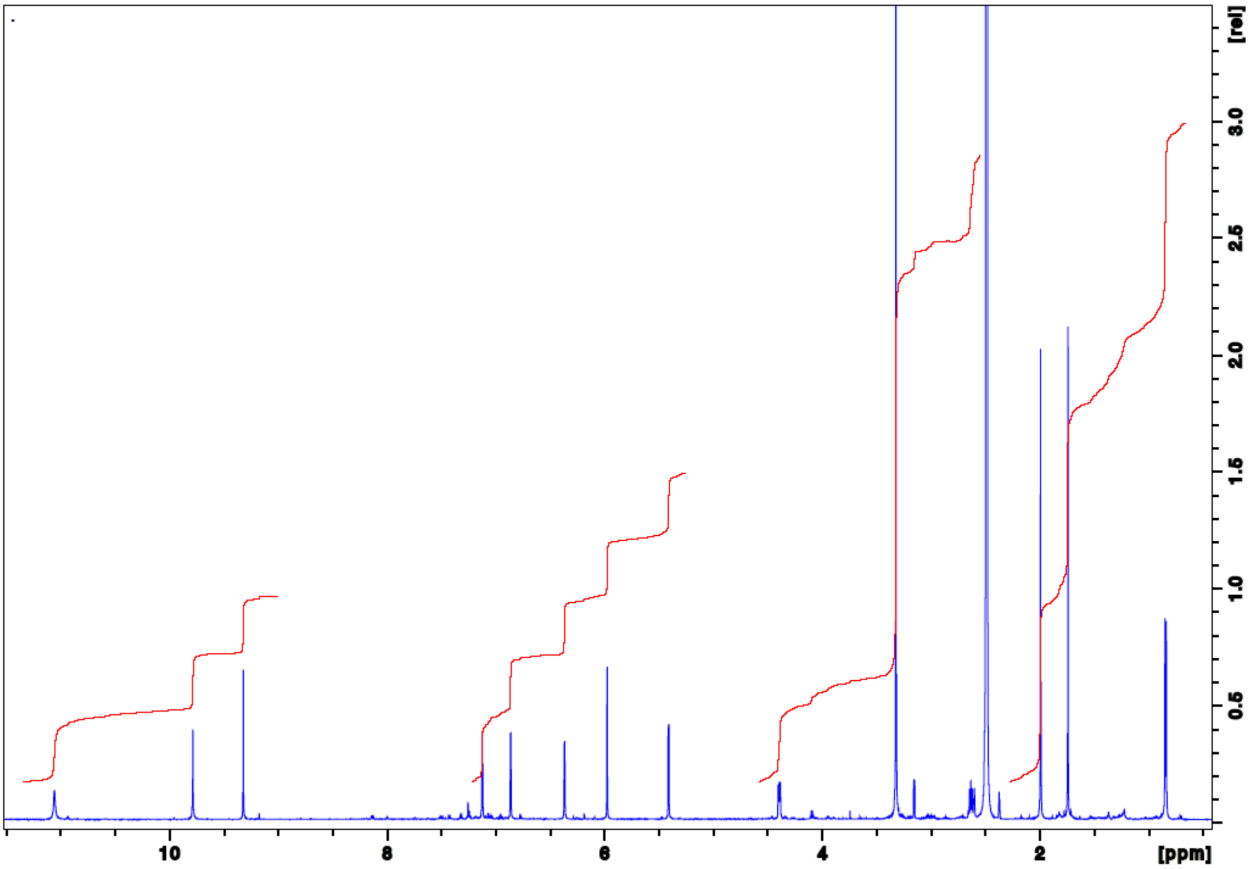

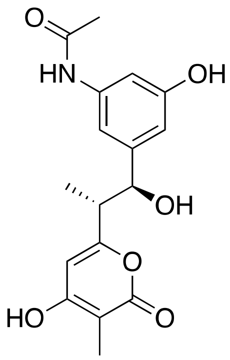

4

Expanded ^{1}H NMR Spectrum of Salinacetamide (**4**) with peak picking recorded at 600 MHz in DMSO-d_6

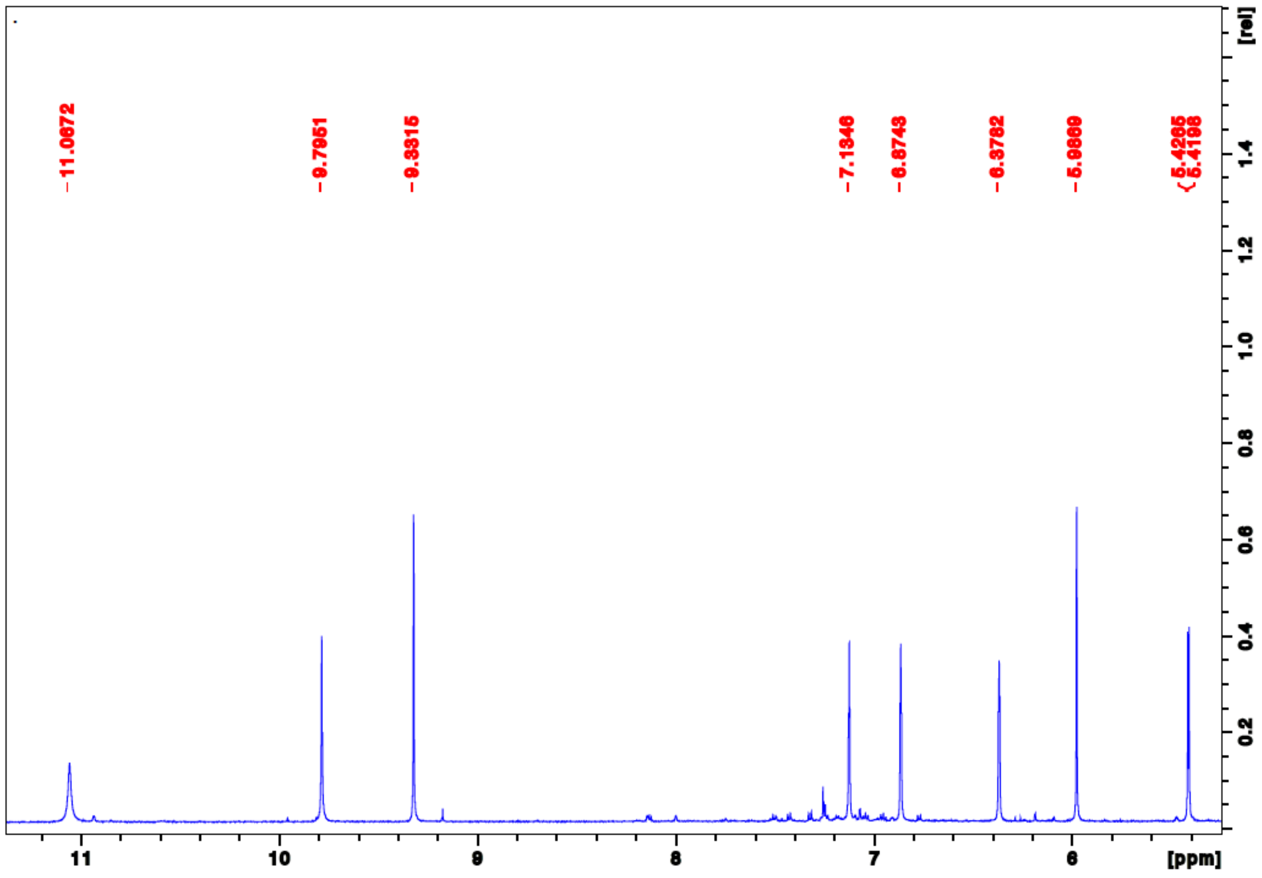

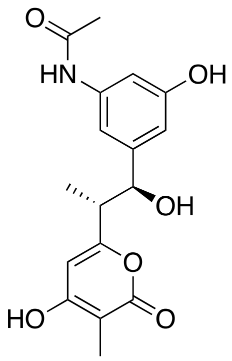

4

Expanded ^{1}H NMR Spectrum of Salinacetamide (**4**) with peak picking recorded at 600 MHz in DMSO-d_6

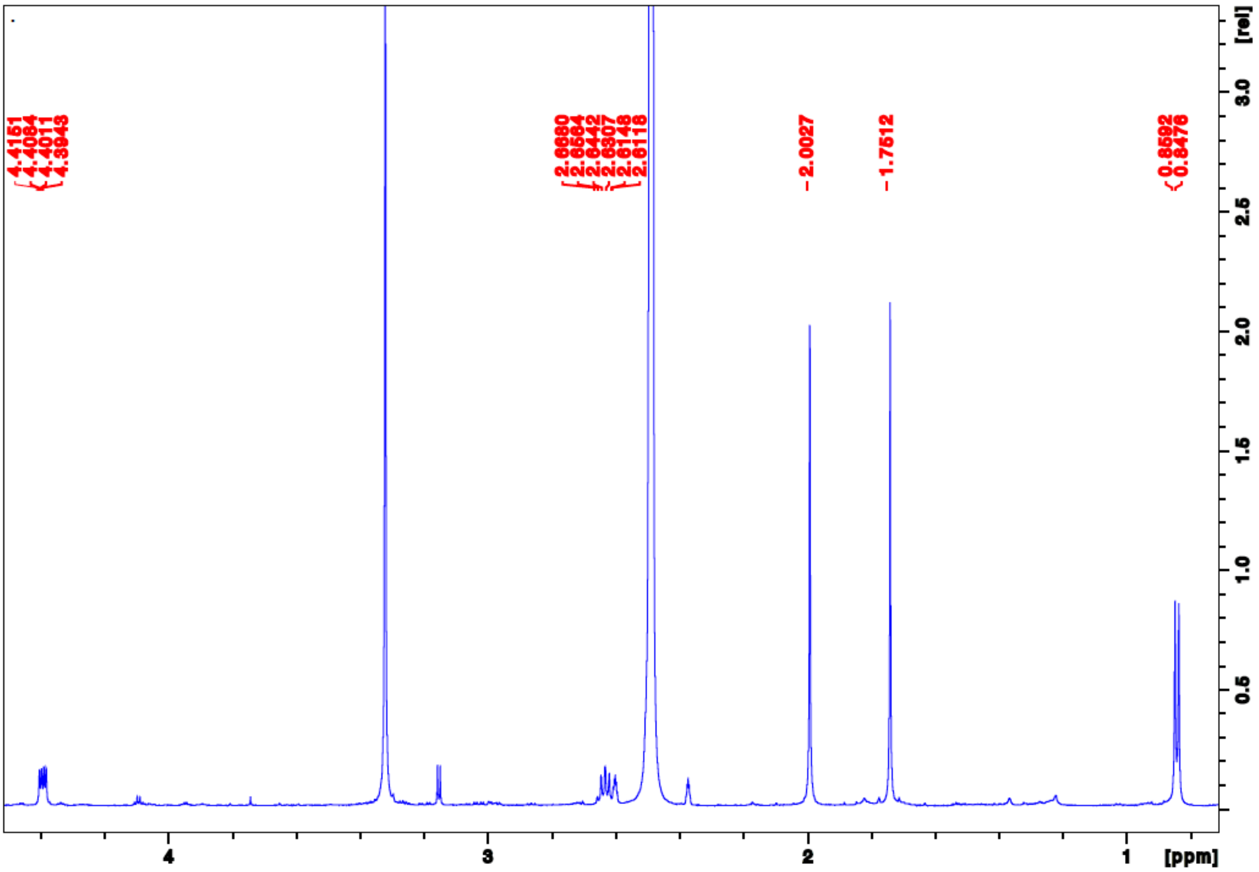

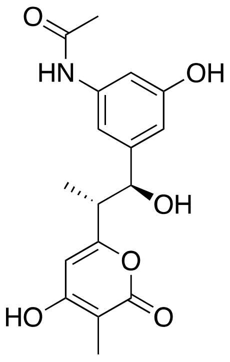

4

^{13}C NMR Spectrum of Salinacetamide (**4**) recorded at 150 MHz in DMSO-d_6

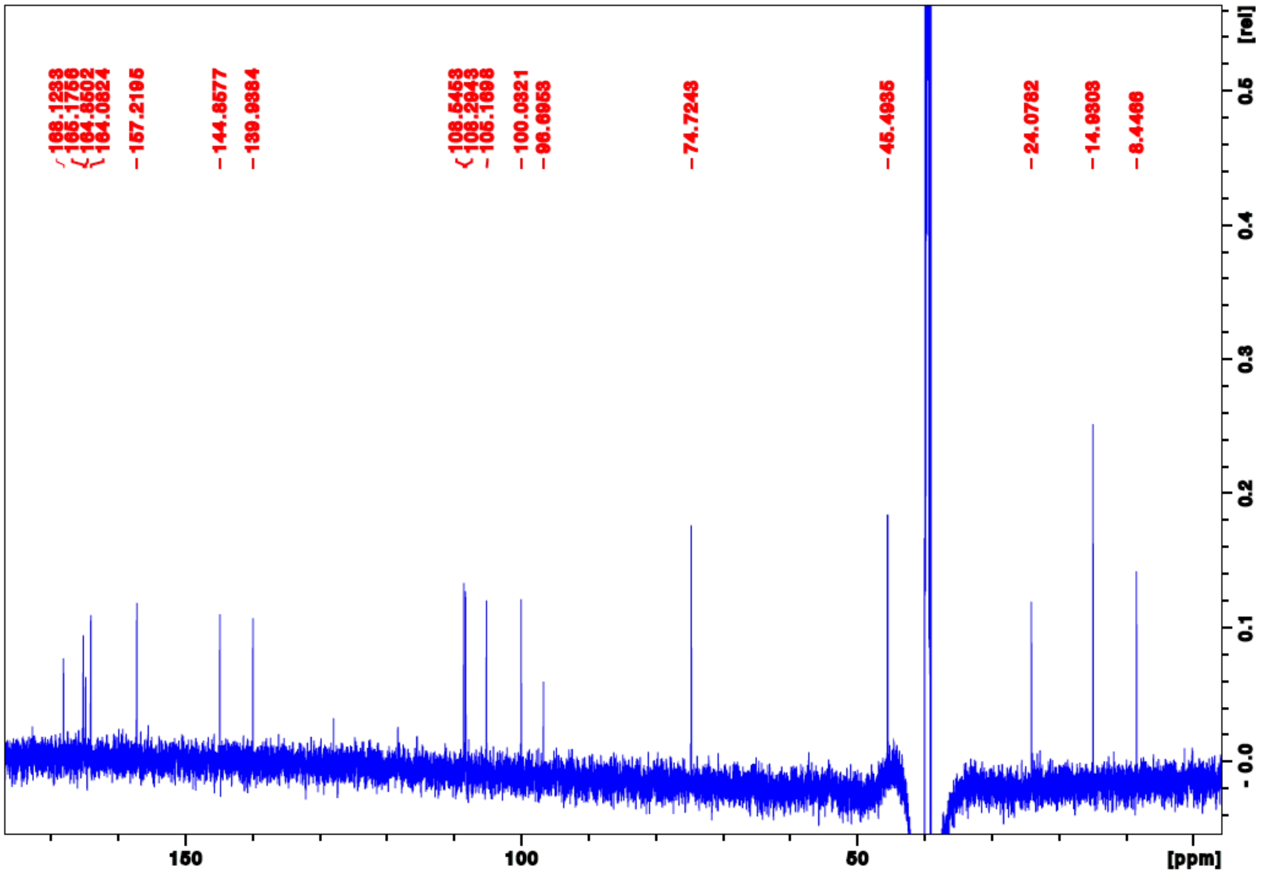

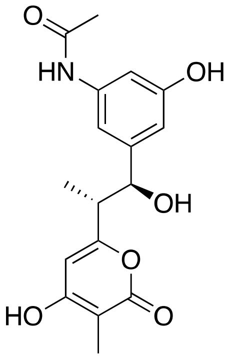

4

gradCOSY60 NMR Spectrum of Salinacetamide (**4**) recorded at 600 MHz in DMSO-d_6

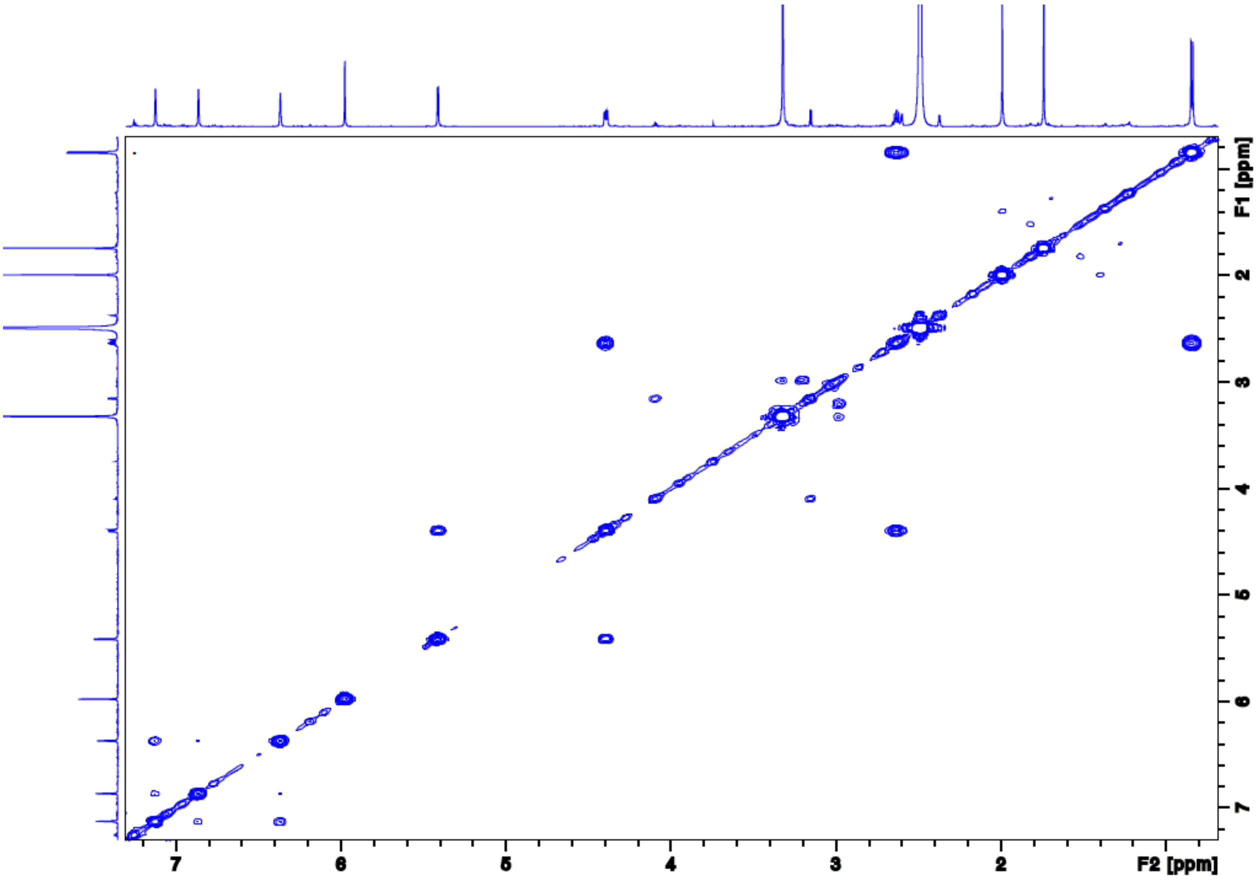

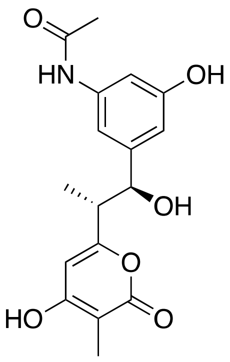

4

gradHSQC NMR Spectrum of Salinacetamide (**4**) recorded at 600 MHz in DMSO-d_6

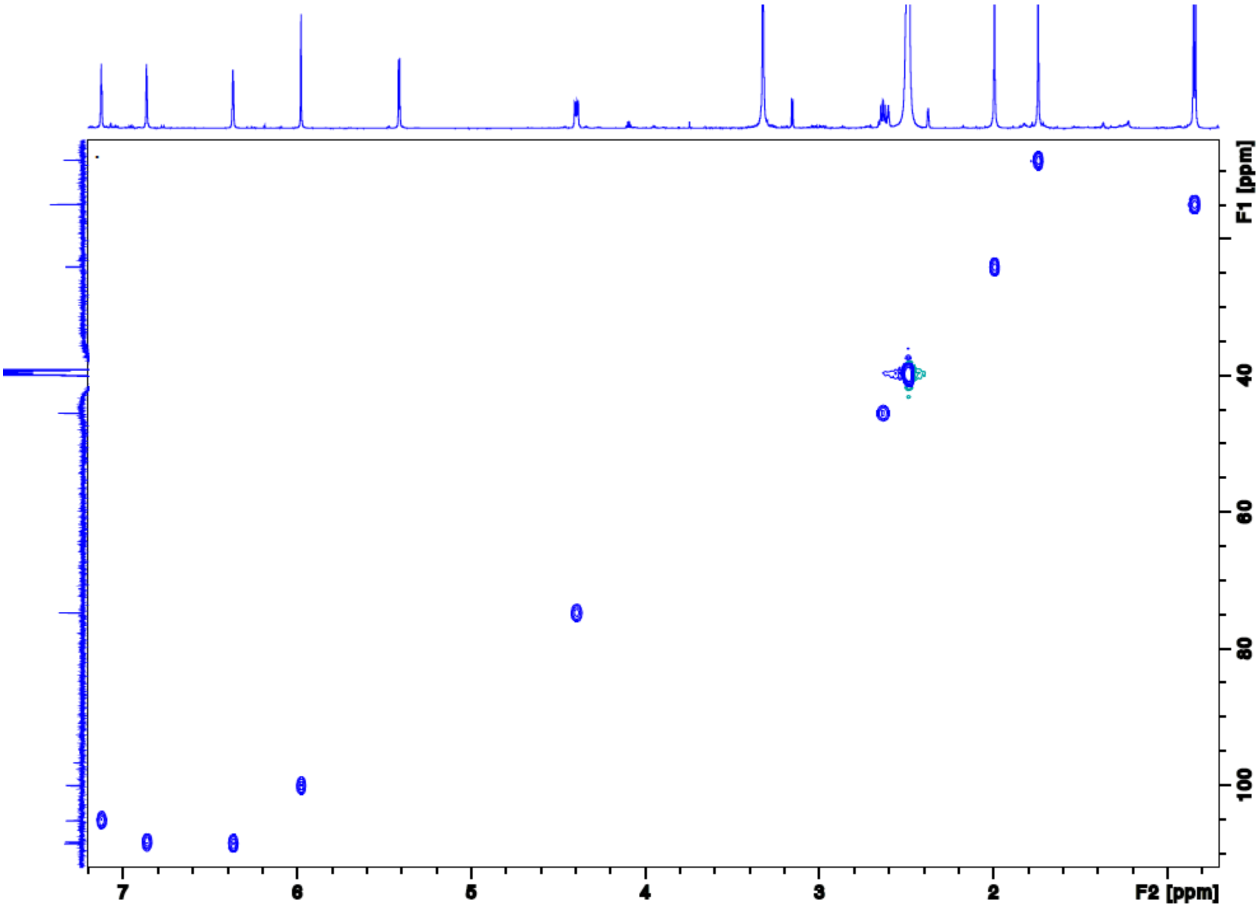

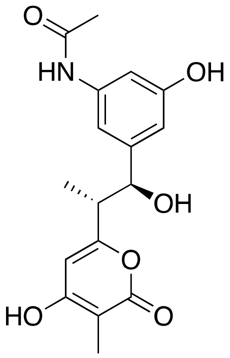

4

gradHMBC NMR Spectrum of Salinacetamide (**4**) recorded at 600 MHz in DMSO-d_6

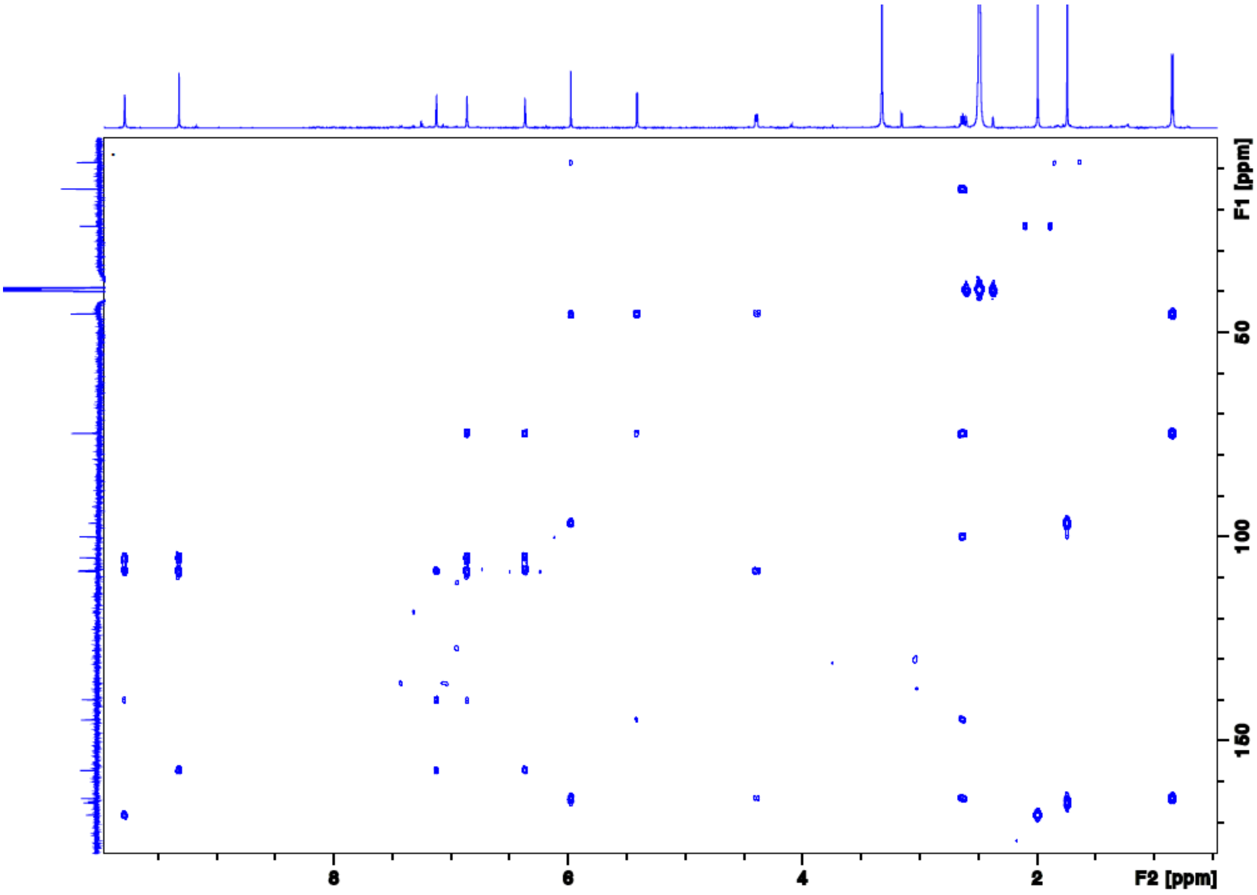

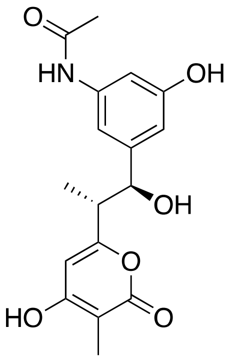

4

tROESY NMR Spectrum of Salinacetamide (**4**) recorded at 600 MHz in DMSO-d_6

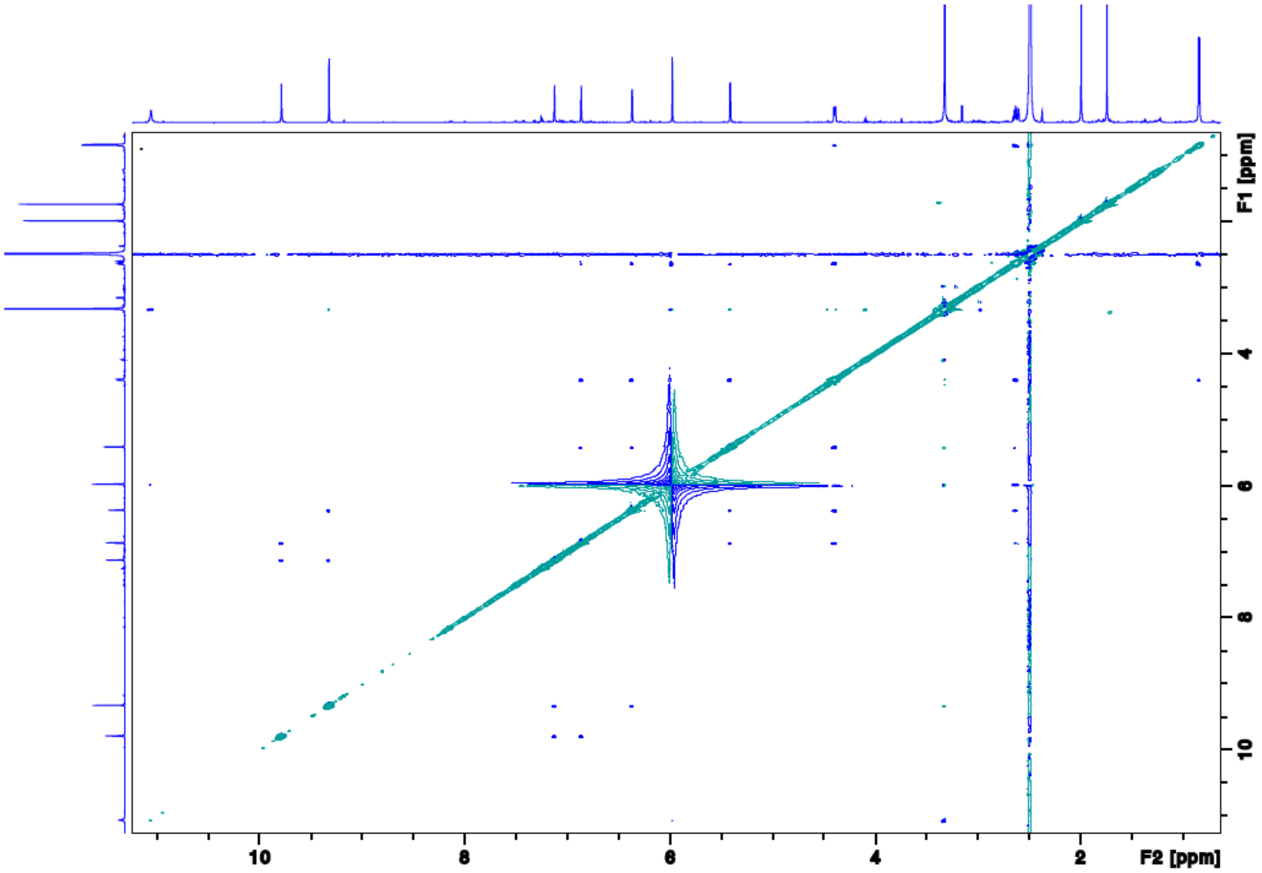

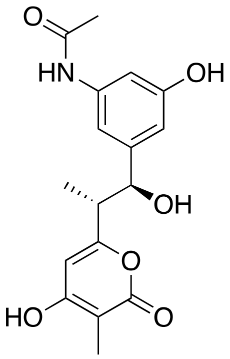

4

grad^{15}N-HSQC NMR Spectrum of Salinacetamide (**4**) recorded at 600 MHz in DMSO-d_6

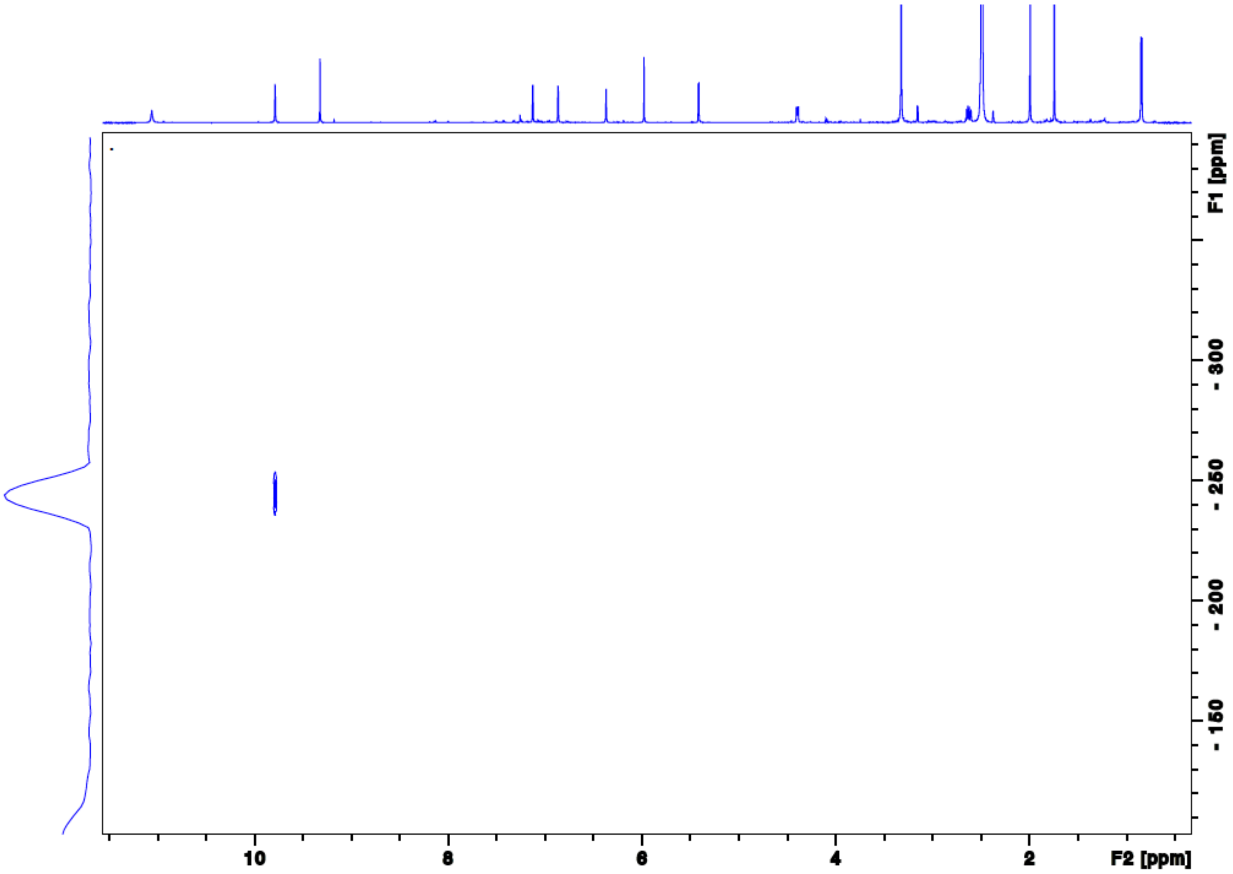

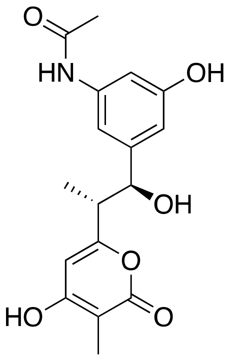

4

^{1}H NMR Spectrum of Salinisporamine (**5**) in the presence of TFA recorded at 600 MHz in DMSO-d_6

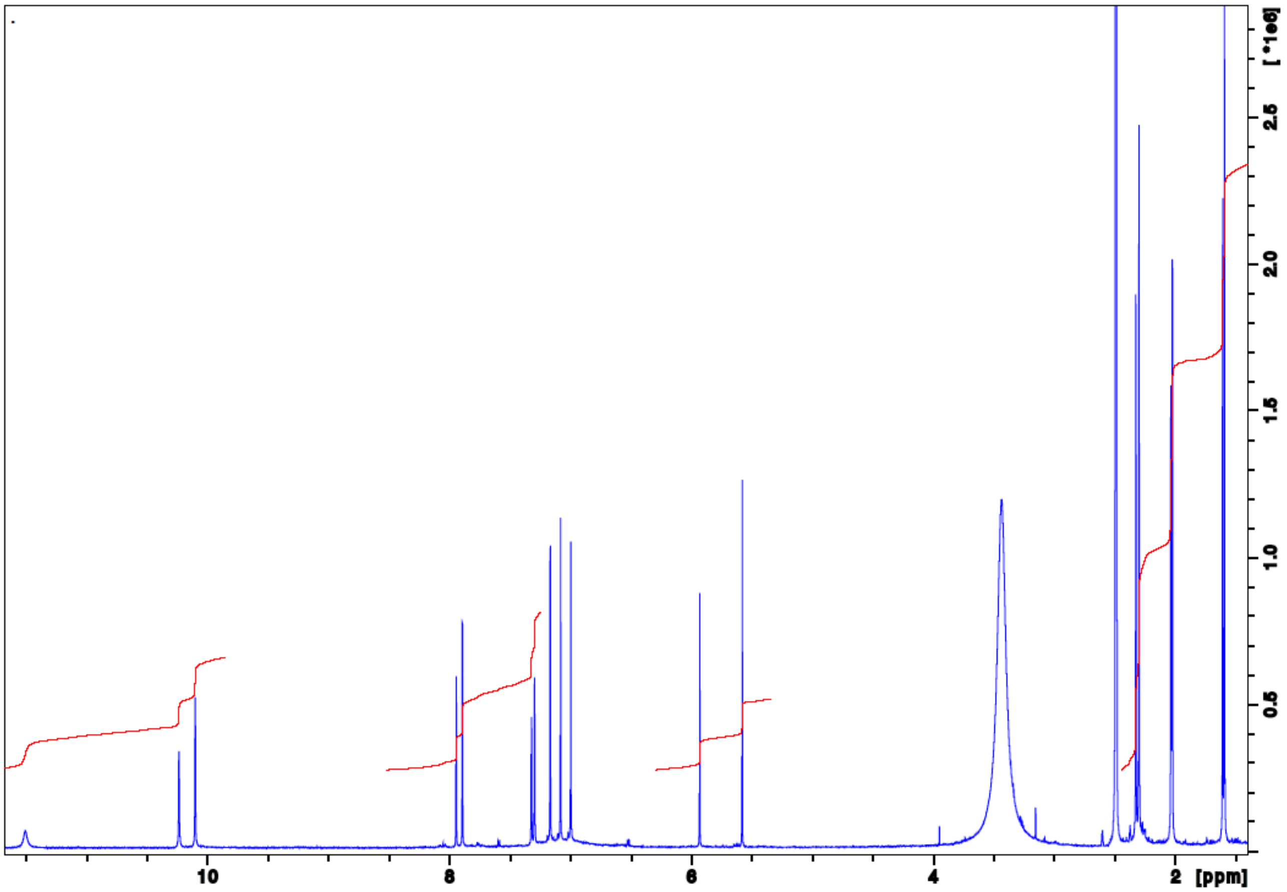

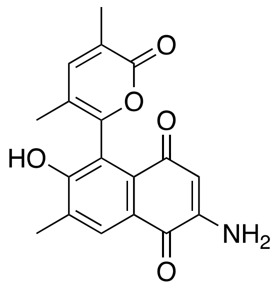

5

^{1}H NMR Spectrum of Salinisporamine (**5**) recorded at 600 MHz in DMSO-d_6

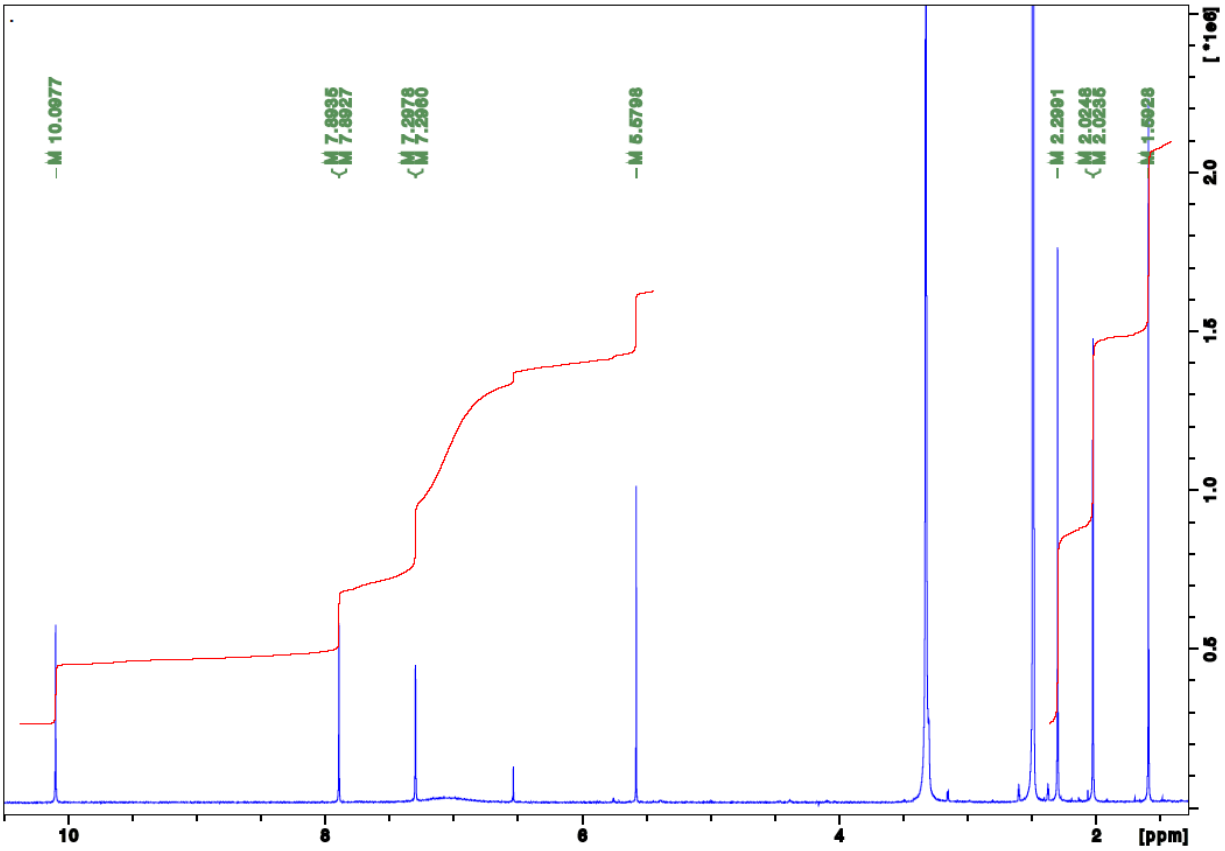

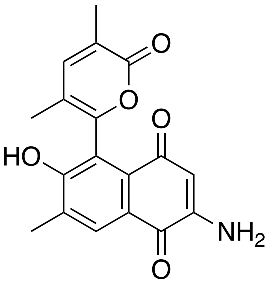

5

^{13}C NMR Spectrum of Salinisporamine (**5**) recorded at 150 MHz in DMSO-d_6

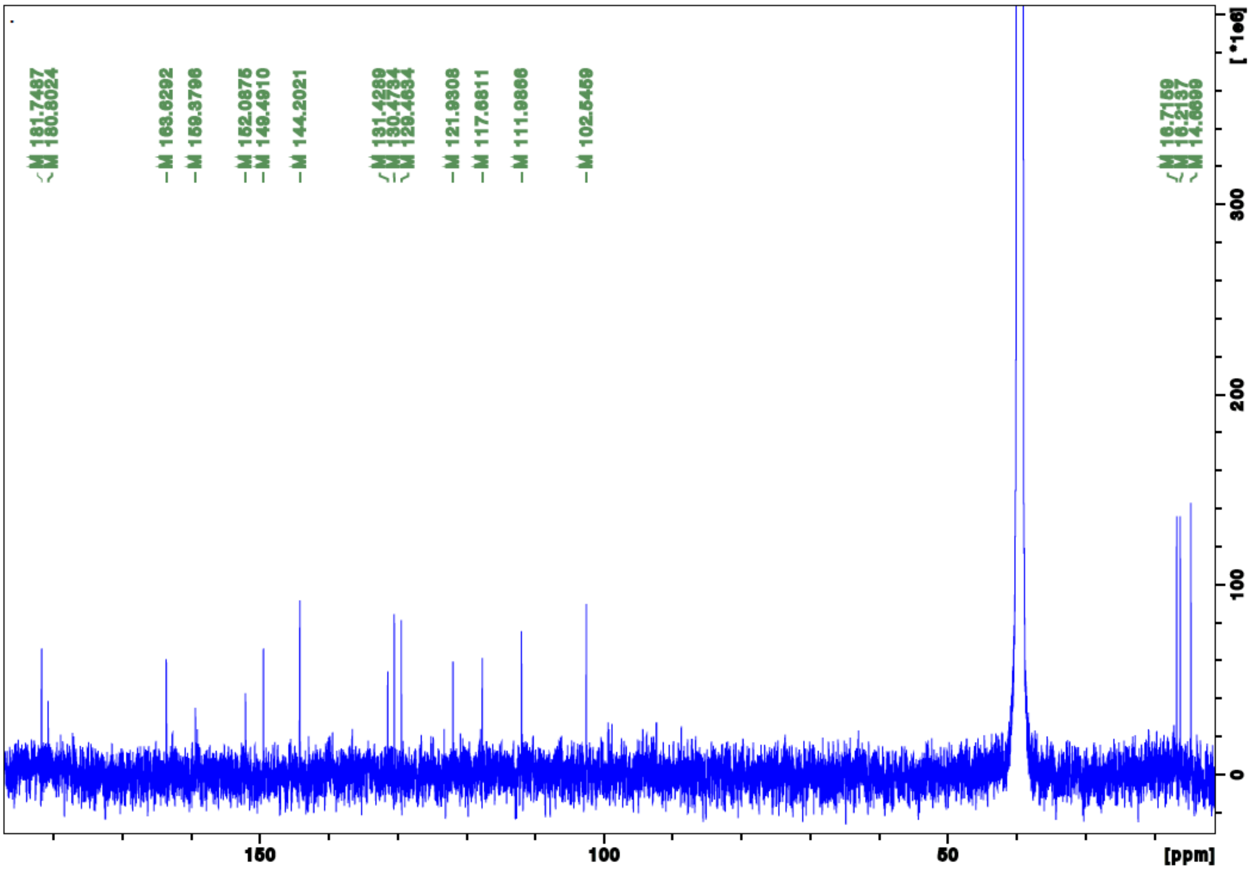

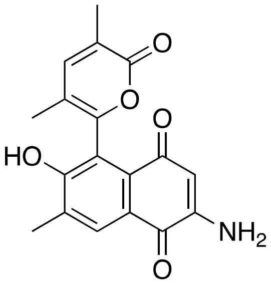

5

gradCOSY60 NMR Spectrum of Salinisporamine (**5**) recorded at 600 MHz in DMSO-d_6

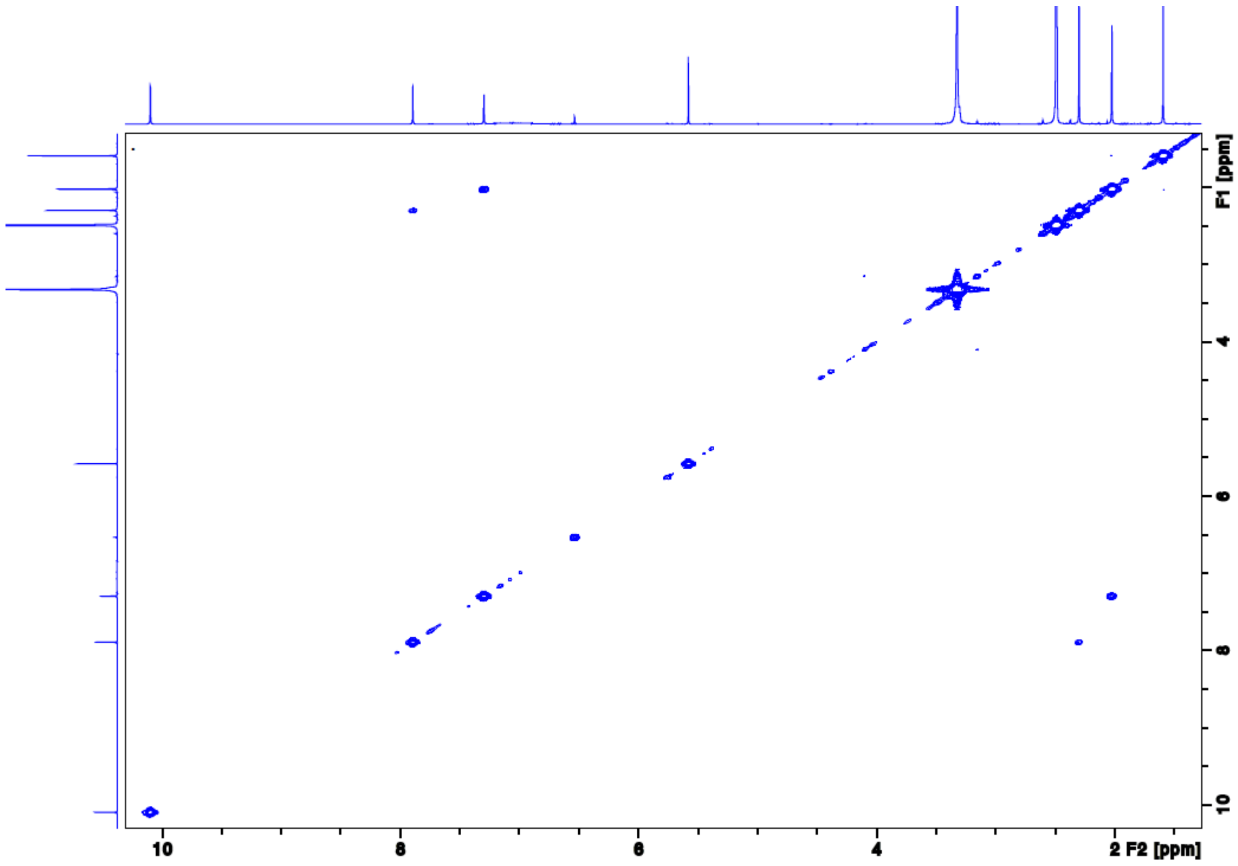

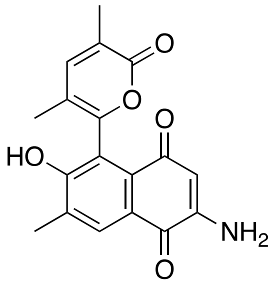

5

gradHSQC NMR Spectrum of Salinisporamine (**5**) recorded at 600 MHz in DMSO-d_6

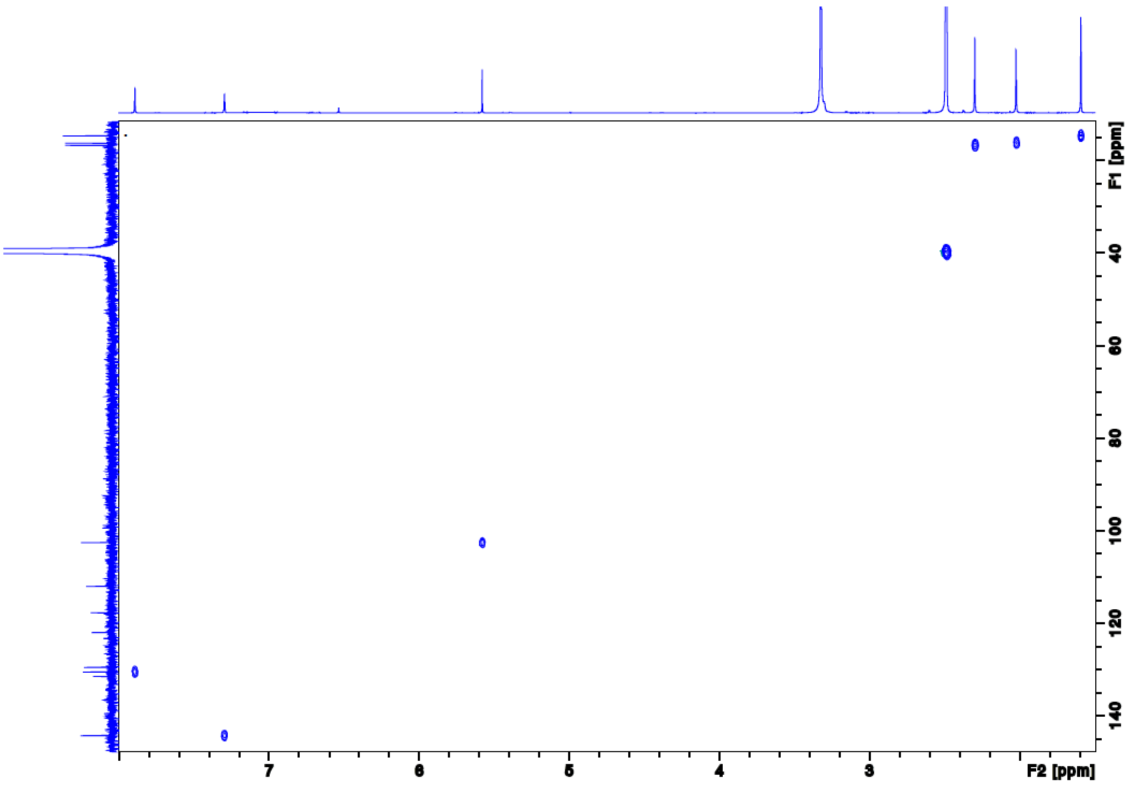

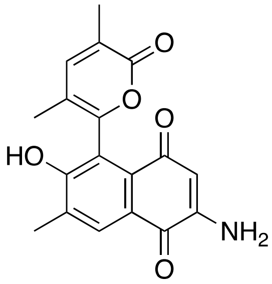

5

gradHMBC NMR Spectrum of Salinisporamine (**5**) recorded at 600 MHz in DMSO-d_6

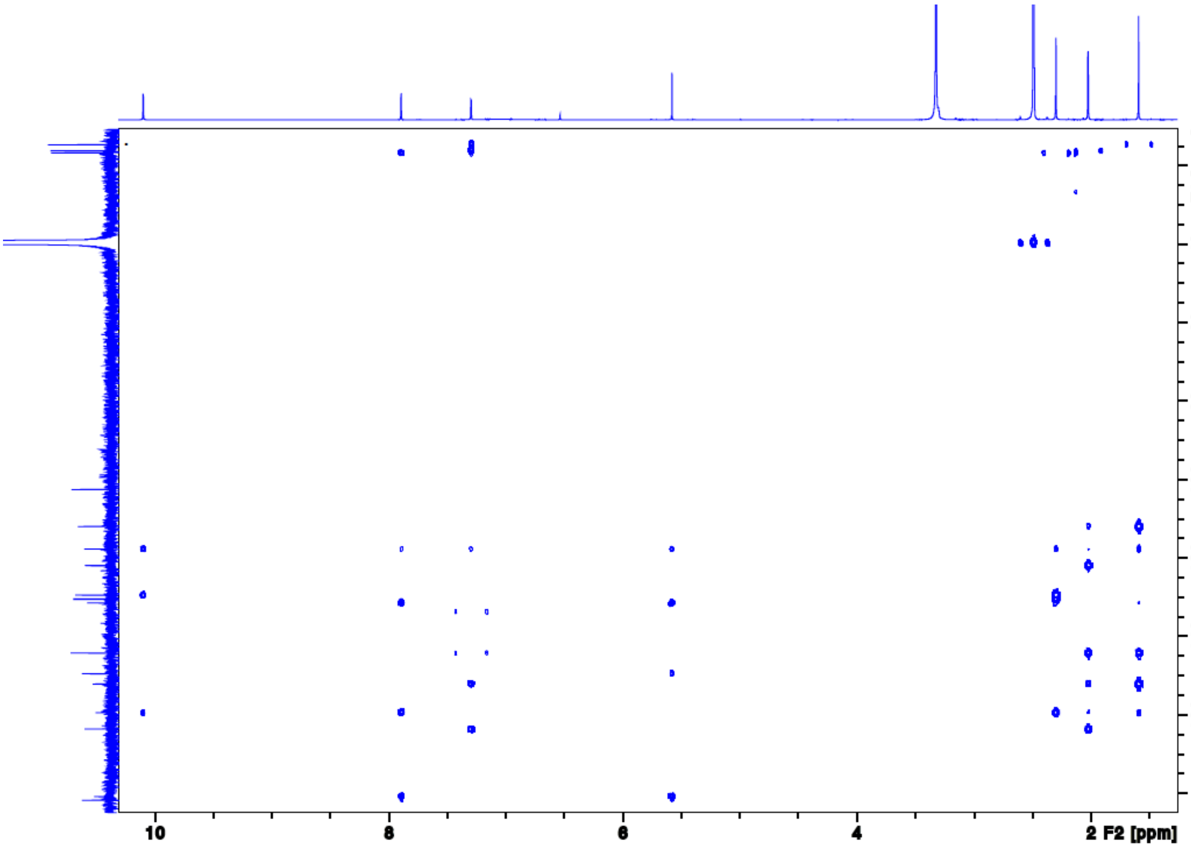

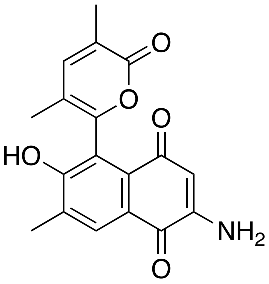

5

tROESY NMR Spectrum of Salinisporamine (**5**) recorded at 600 MHz in DMSO-d_6

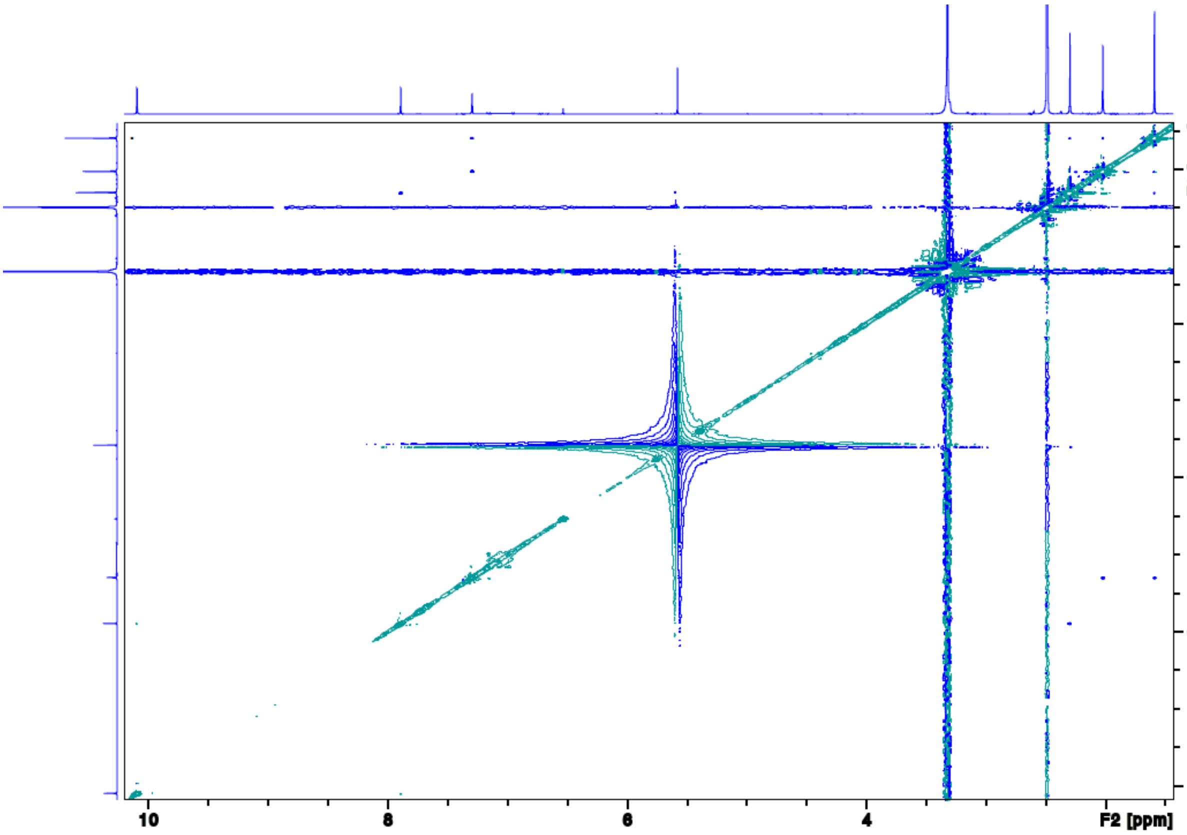

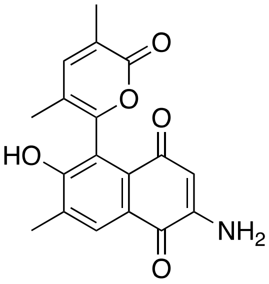

5

Details of the X-ray Diffraction Analysis of Salinisporamine (5).

Crystal Data and Experimental

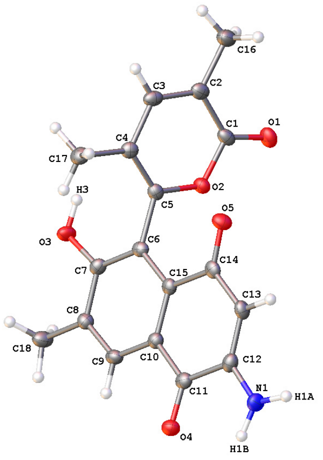

Experimental. Single orange blade-shaped crystals of salinisporamine (**5**) were recrystallised from methanol by slow evaporation. A suitable crystal (0.25×0.10×0.03) mm^3 was selected and mounted on a mylar loop with oil on a Bruker APEX II area detector diffractometer. The crystal was kept at T = 90(2) K during data collection. Using **Olex2** (Dolomanov et al., 2009), the structure was solved with the XT (Sheldrick, 2015) structure solution program, using the Intrinsic Phasing solution method. The model was refined with version 2017/1 of **XL** (Sheldrick, 2015) using Least Squares minimisation.

Crystal Data. $C_{18}H_{15}NO_5$, M_r = 325.31, monoclinic, $P2_1/c$ (No. 14), a = 13.7206(9) Å, b = 6.7934(5) Å, c = 16.8142(11) Å, β = 108.156(4)°, $\alpha = \gamma$ = 90°, V = 1489.21(18) Å^3, T = 90(2) K, Z = 4, Z' = 1, $\mu(CuK_\alpha)$ = 0.892, 9005 reflections measured, 2604 unique (R_{int} = 0.0366) which were used in all calculations. The final wR_2 was 0.1092 (all data) and R_1 was 0.0403 (I > 2(I)).

**Compound**	Salinisporamine (**5**)
Formula	$C_{18}H_{15}NO_5$
$D_{calc.}$/ g cm^{-3}	1.451
μ/mm^{-1}	0.892
Formula Weight	325.31
Colour	orange
Shape	blade
Size/mm^3	0.25×0.10×0.03
T/K	90(2)
Crystal System	monoclinic
Space Group	$P2_1/c$
a/Å	13.7206(9)
b/Å	6.7934(5)
c/Å	16.8142(11)
α/°	90
β/°	108.156(4)
γ/°	90
V/Å^3	1489.21(18)
Z	4
Z'	1
Wavelength/Å	1.54178
Radiation type	CuK_α
Θ_{min}/°	3.390
Θ_{max}/°	66.735
Measured Refl.	9005
Independent Refl.	2604
Reflections Used	2030
R_{int}	0.0366
Parameters	232
Restraints	0
Largest Peak	0.214
Deepest Hole	-0.270
GooF	1.041
wR_2 (all data)	0.1092
wR_2	0.1006
R_1 (all data)	0.0563
R_1	0.0403

Structure Quality Indicators

**Reflections:**	d min (Cu) 0.84	I/σ 14.8	Rint 3.66%	complete 99%
**Refinement:**	Shift 0.000	Max Peak 0.2	Min Peak -0.3	GooF 1.041

An orange blade-shaped crystal with dimensions 0.25×0.10×0.03 mm^3 was mounted on a mylar loop with oil. X-ray diffraction data were collected using a Bruker APEX II area detector diffractometer equipped with an Oxford Cryosystems low-temperature device, operating at T = 90(2) K.

Data were measured using and ω scans of 2 ° per frame for 30 s using CuK_α radiation (microfocus sealed X-ray tube, 45 kV, 0.60 mA). The total number of runs and images was based on the strategy calculation from the program APEX2. The maximum resolution achieved was Θ = 66.735°.

Cell parameters were retrieved using the **SAINT** (Bruker, V8.34A, after 2013) software and refined using **SAINT** (Bruker, V8.34A, after 2013) on 4576 reflections, 51 % of the observed reflections. Data reduction was performed using the **SAINT** (Bruker, V8.34A, after 2013) software which corrects for Lorentz polarisation. The final completeness is 98.80 % out to 66.735° in Θ.

A multi-scan absorption correction was performed using SADABS-2014/5 (Bruker, 2014/5) was used for absorption correction. wR_2(int) was 0.0586 before and 0.0480 after correction. The ratio of minimum to maximum transmission is 0.8996. The λ/2 correction factor is 0.00150. The absorption coefficient μ of this material is 0.892 mm^{-1} at this wavelength (λ = 1.54178Å) and the minimum and maximum transmissions are 0.876 and 0.974.

The structure was solved in the space group P2₁/c (# 14) by Intrinsic Phasing using the XT (Sheldrick, 2015) structure solution program and refined by Least Squares using version 2017/1 of **XL** (Sheldrick, 2015). All non-hydrogen atoms were refined anisotropically. All O—H and N—H hydrogen atoms were located in difference maps and refined isotropically. All other hydrogen atom positions were calculated geometrically and refined using the riding model.

exptl_absorpt_process_details: SADABS-2014/5 (Bruker, 2014/5) was used for absorption correction. wR_2(int) was 0.0586 before and 0.0480 after correction. The ratio of minimum to maximum transmission is 0.8996. The λ/2 correction factor is 0.00150.

Table S1: Fractional Atomic Coordinates (×10^4) and Equivalent Isotropic Displacement Parameters (Å^2×10^3) for **ra144**. U_{eq} is defined as 1/3 of the trace of the orthogonalised U_{ij}.

Atom	x	y	z	U_{eq}
O1	195.5(11)	8162(2)	1447.8(9)	23.9(4)
O2	1275.9(11)	9431(2)	2587.1(8)	18.3(3)
O3	1502.0(11)	11563(2)	4645.9(9)	21.9(3)
O4	5600.7(11)	6756(2)	5321.3(9)	22.2(3)
O5	3265.4(11)	9828(2)	2429.6(8)	22.5(4)
N1	6093.0(15)	5936(3)	3952.6(12)	23.6(4)
C1	581.3(16)	9678(3)	1805.8(12)	19.8(4)
C2	369.8(16)	11658(3)	1497.1(12)	21.0(5)
C3	864.8(16)	13158(3)	1987.8(13)	20.6(5)
C4	1623.7(15)	12853(3)	2786.0(12)	18.1(4)
C5	1811.9(15)	10984(3)	3048.4(12)	16.8(4)
C6	2545.1(16)	10246(3)	3842.6(12)	17.1(4)

Atom	x	y	z	U_{eq}
C7	2315.4(16)	10510(3)	4589.1(13)	18.0(4)
C8	2916.1(16)	9657(3)	5346.0(12)	18.6(4)
C9	3786.3(16)	8641(3)	5342.3(12)	19.3(5)
C10	4054.7(15)	8410(3)	4613.2(12)	17.3(4)
C11	5009.3(16)	7359(3)	4659.5(13)	19.2(5)
C12	5254.0(16)	7022(3)	3860.0(13)	20.2(5)
C13	4654.5(16)	7843(3)	3131.6(12)	20.0(5)
C14	3760.2(16)	8992(3)	3084.6(12)	18.7(4)
C15	3437.1(16)	9201(3)	3855.1(12)	16.9(4)
C16	-381.9(18)	11955(3)	643.7(13)	28.5(5)
C17	2209.1(17)	14568(3)	3266.2(13)	24.0(5)
C18	2605.3(17)	9831(3)	6123.1(13)	25.5(5)

Table S2: Anisotropic Displacement Parameters (×10^4) **ra144**. The anisotropic displacement factor exponent takes the form: $-2\pi^2[h^2a^{*2} \times U_{11} + ... + 2hka^* \times b^* \times U_{12}]$

Atom	U_{11}	U_{22}	U_{33}	U_{23}	U_{13}	U_{12}
O1	18.1(8)	28.7(8)	22.8(7)	-6.8(6)	3.1(6)	-4.7(6)
O2	15.5(8)	20.4(8)	17.1(7)	-0.3(6)	2.5(6)	-1.8(6)
O3	17.1(8)	27.9(8)	20.4(7)	3.1(6)	5.5(6)	7.8(6)
O4	19.2(8)	23.7(8)	21.1(7)	0.6(6)	2.6(6)	2.0(6)
O5	20.9(8)	29.0(8)	17.7(7)	3.7(6)	6.0(6)	0.8(6)
N1	21.8(11)	27.4(10)	23(1)	-1.7(8)	8.8(9)	5.7(8)
C1	14.3(11)	29.7(12)	15.2(10)	-0.9(9)	4.0(8)	-0.2(9)
C2	16.0(11)	28.8(12)	19.6(10)	1.2(9)	7.5(9)	3.7(9)
C3	16.9(11)	25.1(11)	21.9(10)	5.8(9)	9.0(9)	5.4(9)
C4	14.1(11)	20.7(11)	20.5(10)	1.3(8)	6.9(9)	-0.6(8)
C5	12.8(11)	20.6(10)	17.5(10)	-1.4(8)	5.4(8)	-1.6(8)
C6	15.7(11)	15.3(10)	18.9(10)	0.7(8)	3.5(9)	-3.4(8)
C7	13.4(10)	18.2(10)	22.4(10)	-0.5(8)	5.3(9)	-0.4(8)
C8	18.7(11)	19.4(11)	18(1)	0.4(8)	6.0(9)	-1.1(8)
C9	17.1(11)	18.7(11)	18.9(10)	1.3(8)	0.9(9)	-0.3(8)
C10	15.8(11)	16.5(10)	19(1)	-1.3(8)	4.6(8)	-1.6(8)
C11	18.1(11)	15.8(10)	22.1(11)	-0.8(8)	3.8(9)	-2.9(8)
C12	17.8(11)	18.6(11)	25.1(11)	-4.2(8)	8.0(9)	-1.5(8)
C13	18.7(11)	24.5(11)	18.3(10)	-4.6(8)	7.9(9)	-2.3(9)
C14	17.7(11)	17.6(11)	19.4(10)	-3.6(8)	3.8(9)	-6.4(8)
C15	15.7(11)	15.8(10)	18.4(10)	-2.9(8)	4.2(9)	-3.4(8)
C16	27.7(13)	33.7(13)	21.2(11)	0.3(9)	3.5(10)	8.4(10)
C17	25.5(13)	18.4(11)	26.4(11)	1.9(9)	5.6(10)	-0.7(9)
C18	23.5(12)	32.9(13)	20.7(11)	5.3(9)	7.9(10)	6.7(10)

Table S3: Bond Lengths in Å for **ra144**.

Atom	Atom	Length/Å
O1	C1	1.227(2)
O2	C1	1.372(2)
O2	C5	1.378(2)
O3	C7	1.354(2)
O4	C11	1.225(2)
O5	C14	1.237(2)
N1	C12	1.335(3)
C1	C2	1.439(3)
C2	C3	1.353(3)
C2	C16	1.497(3)
C3	C4	1.434(3)
C4	C5	1.343(3)
C4	C17	1.500(3)

Atom	Atom	Length/Å
C5	C6	1.487(3)
C6	C7	1.397(3)
C6	C15	1.409(3)
C7	C8	1.407(3)
C8	C9	1.381(3)
C8	C18	1.500(3)
C9	C10	1.394(3)
C10	C11	1.472(3)
C10	C15	1.399(3)
C11	C12	1.501(3)
C12	C13	1.364(3)
C13	C14	1.436(3)
C14	C15	1.501(3)

Table S4: Bond Angles in ° for **ra144**.

Atom	Atom	Atom	Angle/°
C1	O2	C5	122.34(16)
O1	C1	O2	115.61(18)
O1	C1	C2	126.90(19)
O2	C1	C2	117.48(18)
C1	C2	C16	118.24(19)
C3	C2	C1	118.54(19)
C3	C2	C16	123.2(2)
C2	C3	C4	122.8(2)
C3	C4	C17	120.15(18)
C5	C4	C3	116.95(19)
C5	C4	C17	122.80(19)
O2	C5	C6	109.89(16)
C4	C5	O2	121.65(18)
C4	C5	C6	128.41(18)
C7	C6	C5	118.99(18)
C7	C6	C15	119.27(18)
C15	C6	C5	121.65(18)
O3	C7	C6	123.54(18)
O3	C7	C8	114.64(18)
C6	C7	C8	121.80(19)

Atom	Atom	Atom	Angle/°
C7	C8	C18	120.66(19)
C9	C8	C7	117.71(18)
C9	C8	C18	121.62(19)
C8	C9	C10	121.72(19)
C9	C10	C11	118.84(18)
C9	C10	C15	120.45(19)
C15	C10	C11	120.70(18)
O4	C11	C10	122.45(19)
O4	C11	C12	119.51(19)
C10	C11	C12	118.05(18)
N1	C12	C11	113.92(19)
N1	C12	C13	125.99(19)
C13	C12	C11	120.07(19)
C12	C13	C14	122.54(19)
O5	C14	C13	121.41(19)
O5	C14	C15	119.83(19)
C13	C14	C15	118.75(18)
C6	C15	C14	121.55(18)
C10	C15	C6	118.92(18)
C10	C15	C14	119.48(18)

Table S5: Torsion Angles in ° for **ra144**.

Atom	Atom	Atom	Atom	Angle/°
O1	C1	C2	C3	179.2(2)
O1	C1	C2	C16	-1.3(3)
O2	C1	C2	C3	0.2(3)
O2	C1	C2	C16	179.67(17)
O2	C5	C6	C7	-104.7(2)
O2	C5	C6	C15	72.0(2)
O3	C7	C8	C9	-176.95(18)
O3	C7	C8	C18	3.9(3)
O4	C11	C12	N1	4.5(3)
O4	C11	C12	C13	-173.8(2)
O5	C14	C15	C6	3.5(3)
O5	C14	C15	C10	-173.90(19)
N1	C12	C13	C14	179.9(2)
C1	O2	C5	C4	5.4(3)
C1	O2	C5	C6	-176.79(16)
C1	C2	C3	C4	2.8(3)
C2	C3	C4	C5	-1.9(3)
C2	C3	C4	C17	174.7(2)
C3	C4	C5	O2	-2.2(3)
C3	C4	C5	C6	-179.58(19)
C4	C5	C6	C7	72.9(3)
C4	C5	C6	C15	-110.4(2)
C5	O2	C1	O1	176.58(17)
C5	O2	C1	C2	-4.3(3)
C5	C6	C7	O3	-6.2(3)
C5	C6	C7	C8	172.79(19)
C5	C6	C15	C10	-175.02(18)
C5	C6	C15	C14	7.6(3)
C6	C7	C8	C9	4.0(3)

Atom	Atom	Atom	Atom	Angle/°
C6	C7	C8	C18	-175.16(19)
C7	C6	C15	C10	1.7(3)
C7	C6	C15	C14	-175.72(18)
C7	C8	C9	C10	-1.7(3)
C8	C9	C10	C11	178.23(19)
C8	C9	C10	C15	-0.5(3)
C9	C10	C11	O4	-3.4(3)
C9	C10	C11	C12	176.47(18)
C9	C10	C15	C6	0.5(3)
C9	C10	C15	C14	177.95(18)
C10	C11	C12	N1	-175.39(18)
C10	C11	C12	C13	6.3(3)
C11	C10	C15	C6	-178.17(18)
C11	C10	C15	C14	-0.7(3)
C11	C12	C13	C14	-2.0(3)
C12	C13	C14	O5	175.3(2)
C12	C13	C14	C15	-3.7(3)
C13	C14	C15	C6	-177.56(18)
C13	C14	C15	C10	5.1(3)
C15	C6	C7	O3	177.03(18)
C15	C6	C7	C8	-4.0(3)
C15	C10	C11	O4	175.33(19)
C15	C10	C11	C12	-4.8(3)
C16	C2	C3	C4	-176.61(19)
C17	C4	C5	O2	-178.73(18)
C17	C4	C5	C6	3.9(3)
C18	C8	C9	C10	177.4(2)

Table S6: Hydrogen Fractional Atomic Coordinates (×10^4) and Equivalent Isotropic Displacement Parameters (Å^2×10^3) for **ra144**. U_{eq} is defined as 1/3 of the trace of the orthogonalised U_{ij}.

Atom	x	y	z	U_{eq}
H3	1120(20)	12030(40)	4150(18)	51(9)
H1A	6297(19)	5570(30)	3494(15)	31(7)
H1B	6450(20)	5560(40)	4487(16)	33(7)
H3A	702.67	14469.03	1795.48	25
H9	4213.24	8084.55	5849.68	23
H13	4835.64	7647.06	2636.19	24
H16A	-535.44	13361.41	551.26	43
H16B	-1014.98	11234.83	600.81	43
H16C	-87.24	11464.68	220.44	43
H17A	2531.92	14186.33	3851.47	36
H17B	1738.4	15669.36	3239.48	36
H17C	2739.53	14970.21	3021.27	36
H18A	2537.41	11224.93	6247.18	38
H18B	3128.68	9218.19	6593.29	38
H18C	1946.82	9165.23	6036.6	38

Table S7: Hydrogen Bond information for **ra144**.

D	H	A	d(D-H)/Å	d(H-A)/Å	d(D-A)/Å	D-H-A/deg
O3	H3	O1[1]	0.89(3)	1.94(3)	2.705(2)	144(3)
N1	H1A	O5[2]	0.93(2)	1.90(3)	2.832(2)	179(2)

[1]-x,1/2+y,1/2-z; [2]1-x,-1/2+y,1/2-z

Citations

Dolomanov, O.V.; Bourhis, L.J.; Gildea, R.J.; Howard, J.A.K.; Puschmann, H. Olex2: A complete structure solution, refinement and analysis program, *J. Appl. Cryst.*, **2009**, *42*, 339-341.

Sheldrick, G.M. XT, *Acta Cryst.*, **2015**, *A71*, 3-8.

Sheldrick, G.M. XL, *Acta Cryst.*, **2015**, *C71*, 3-8.

Software for the Integration of CCD Detector System Bruker Analytical X-ray Systems, Bruker axs, Madison, WI (after 2013).

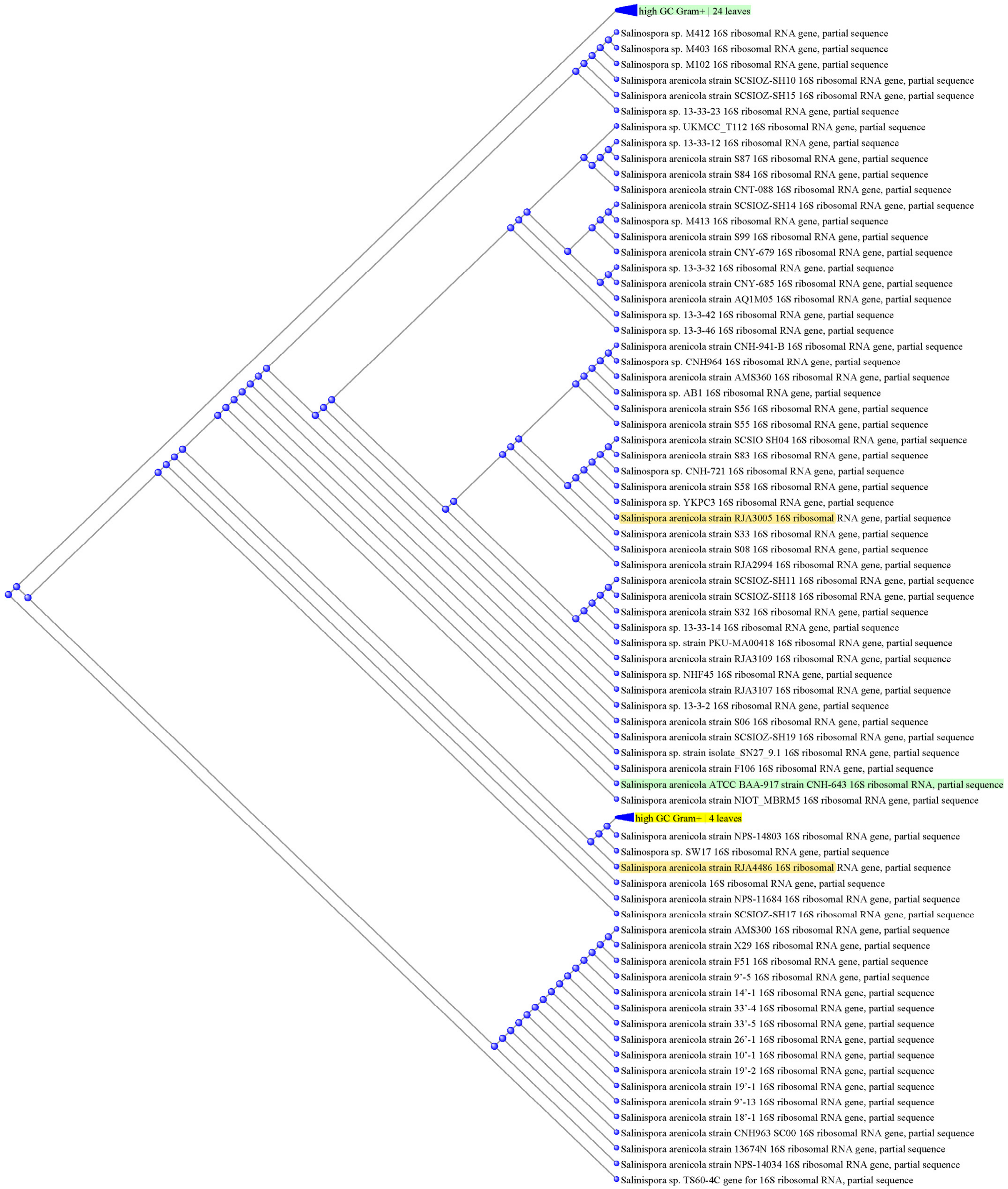

Phylogenetic Tree constructed from all deposited *Salinispora* 16s RNA sequences. The Fast Minimum Evolution Tree Method was used with 0.75 maximum sequence difference.

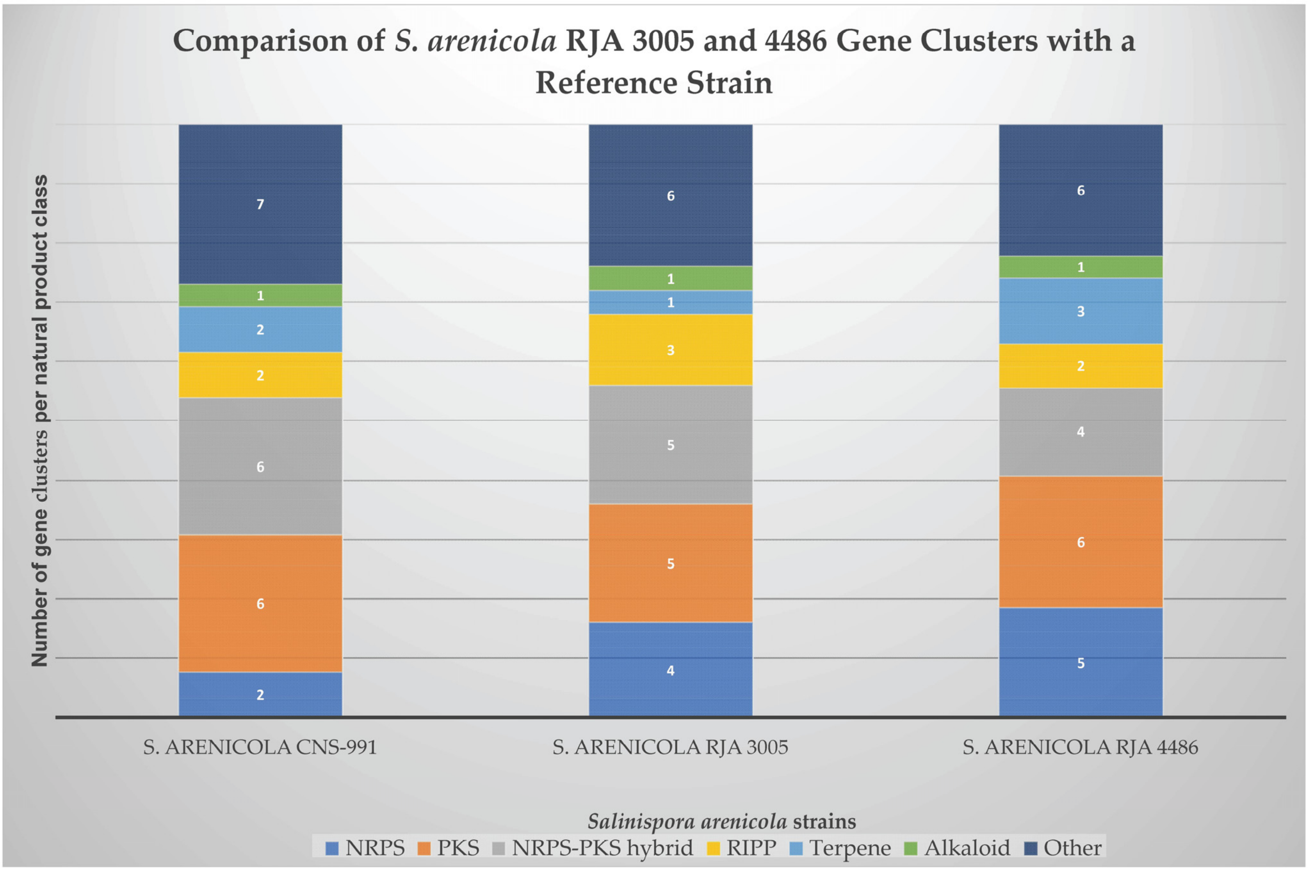
Comparison of S. arenicola RJA 3005 and 4486 Gene Clusters with a Reference Strain
Number of gene clusters per natural product class
7
1
2
2
6
6
2
6
1
1
3
5
5
4
6
1
3
2
4
6
5
S. ARENICOLA CNS-991
S. ARENICOLA RJA 3005
S. ARENICOLA RJA 4486
Salinispora arenicola strains
NRPS
PKS
NRPS-PKS hybrid
RIPP
Terpene
Alkaloid
Other

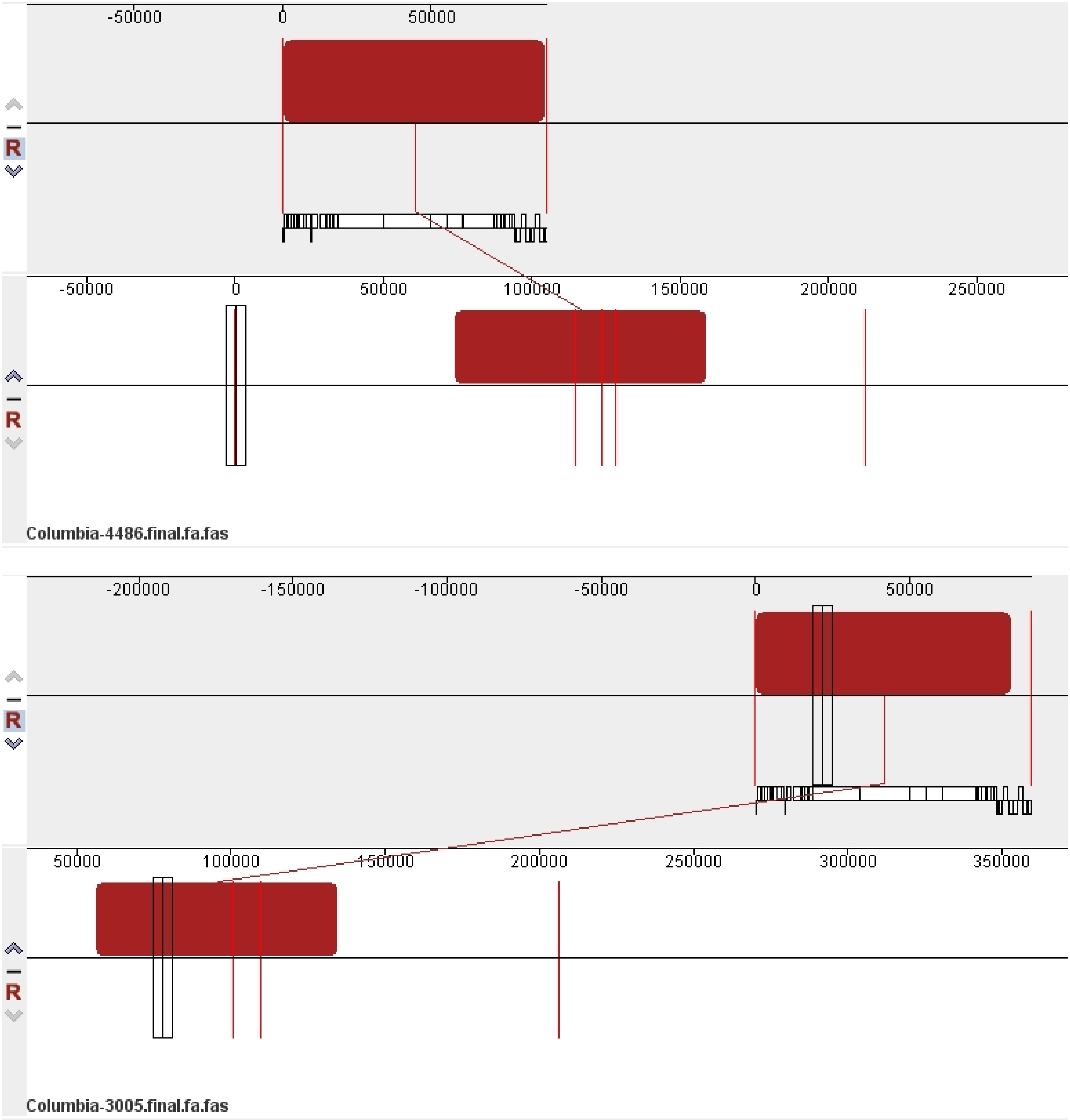

Mauve alignments with the *S. arenicola* CNS-205 rifamycin gene cluster extracted from antiSMASH. The contigs containing partial rifamycin gene clusters of *S. arenicola* RJA 4486 and RJA 3005 were reorganized in alignment with the complete gene cluster.
